# Investigating Abiotic and Biotic Mechanisms of Pyrite Reduction

**DOI:** 10.3389/fmicb.2022.878387

**Published:** 2022-05-09

**Authors:** Rachel L. Spietz, Devon Payne, Gargi Kulkarni, William W. Metcalf, Eric E. Roden, Eric S. Boyd

**Affiliations:** ^1^Department of Microbiology and Cell Biology, Montana State University, Bozeman, MT, United States; ^2^Department of Microbiology, University of Illinois at Urbana-Champaign, Urbana, IL, United States; ^3^Department of Geosciences, University of Wisconsin, Madison, WI, United States

**Keywords:** methanogens, pyrrhotite (Fe_1–_*_*x*_*S), dissolution, hydrogen, extracellular electron transfer, pyrite (FeS_2_)

## Abstract

Pyrite (FeS_2_) has a very low solubility and therefore has historically been considered a sink for iron (Fe) and sulfur (S) and unavailable to biology in the absence of oxygen and oxidative weathering. Anaerobic methanogens were recently shown to reduce FeS_2_ and assimilate Fe and S reduction products to meet nutrient demands. However, the mechanism of FeS_2_ mineral reduction and the forms of Fe and S assimilated by methanogens remained unclear. Thermodynamic calculations described herein indicate that H_2_ at aqueous concentrations as low as 10^–10^ M favors the reduction of FeS_2_, with sulfide (HS^–^) and pyrrhotite (Fe_1–_*_*x*_*S) as products; abiotic laboratory experiments confirmed the reduction of FeS_2_ with dissolved H_2_ concentrations greater than 1.98 × 10^–4^ M H_2_. Growth studies of *Methanosarcina barkeri* provided with FeS_2_ as the sole source of Fe and S resulted in H_2_ production but at concentrations too low to drive abiotic FeS_2_ reduction, based on abiotic laboratory experimental data. A strain of *M. barkeri* with deletions in all [NiFe]-hydrogenases maintained the ability to reduce FeS_2_ during growth, providing further evidence that extracellular electron transport (EET) to FeS_2_ does not involve H_2_ or [NiFe]-hydrogenases. Physical contact between cells and FeS_2_ was required for mineral reduction but was not required to obtain Fe and S from dissolution products. The addition of a synthetic electron shuttle, anthraquinone-2,6-disulfonate, allowed for biological reduction of FeS_2_ when physical contact between cells and FeS_2_ was prohibited, indicating that exogenous electron shuttles can mediate FeS_2_ reduction. Transcriptomics experiments revealed upregulation of several cytoplasmic oxidoreductases during growth of *M. barkeri* on FeS_2_, which may indicate involvement in provisioning low potential electrons for EET to FeS_2_. Collectively, the data presented herein indicate that reduction of insoluble FeS_2_ by *M. barkeri* occurred *via* electron transfer from the cell surface to the mineral surface resulting in the generation of soluble HS^–^ and mineral-associated Fe_1–_*_*x*_*S. Solubilized Fe(II), but not HS^–^, from mineral-associated Fe_1–_*_*x*_*S reacts with aqueous HS^–^ yielding aqueous iron sulfur clusters (FeS_*aq*_) that likely serve as the Fe and S source for methanogen growth and activity. FeS_*aq*_ nucleation and subsequent precipitation on the surface of cells may result in accelerated EET to FeS_2_, resulting in positive feedback between cell activity and FeS_2_ reduction.

## Introduction

Iron disulfide, or pyrite (FeS_2_), is the most abundant sulfide mineral in Earth’s crust and its formation and fate modulate the biogeochemical cycles of iron (Fe), sulfur (S), oxygen, and carbon (Berner, 1984; Benning et al., 2000; Schoonen, 2004). Under oxic conditions, aerobic microorganisms can accelerate the oxidative dissolution of FeS_2_ (Percak-Dennett et al., 2017), a process that represents the primary input of sulfur to both the marine (Konhauser et al., 2011) and terrestrial biospheres since at least 2.8 Gya (Stüeken et al., 2012) or even earlier (Crowe et al., 2013). However, far less is known of the fate of FeS_2_ in anoxic environments. Anaerobic oxidation of FeS_2_ coupled to manganese oxide reduction (Schippers and Jørgensen, 2001) or nitrate reduction (Jorgensen et al., 2009; Jakus et al., 2021) has been demonstrated by microorganisms inhabiting marine sediments and subsurface aquifers, respectively. However, the generation of substantial manganese oxide or nitrate to sustain such reactions requires oxygen (Tipping, 1984; Garvin et al., 2009) or, in the absence of oxygen, abiotic or biotic photochemical processes (Johnson et al., 2013; Daye et al., 2019; Liu et al., 2020). These factors, therefore, likely limit the distribution of anaerobic FeS_2_ oxidation processes, both biotic and abiotic, to near surface environments that are either suboxic to anoxic or where light is available.

In addition to oxidative pathways, studies have shown that FeS_2_ can be abiotically reduced at high temperature (>90°C) and in the presence of high hydrogen (H_2_) partial pressures (>8 bar, equivalent to 7 mM aqueous; Hall, 1986; Truche et al., 2010). During abiotic FeS_2_ reduction at high temperature, sulfide (HS^–^) is released into solution and the iron sulfide mineral pyrrhotite (Fe_1–_*_*x*_*S) precipitates on the surface of FeS_2_ (Truche et al., 2010). At lower temperatures, reduced chromium ions can also promote abiotic reduction of FeS_2_, a feature that is exploited to determine total S in FeS_2_-containing environmental samples (Canfield et al., 1986). However, high temperature (>90°C) environments with high concentrations of H_2_ (>7 mM aqueous) or environments that have high concentrations of reduced chromium ions are rare and, in the case of the former, are likely limited to deep subsurface systems where microbial life is highly restricted or not possible (Colwell and D’Hondt, 2013; Orcutt et al., 2013; Colman et al., 2017; Bradley et al., 2020).

Recently, pure cultures of methanogenic archaea (*Methanosarcina barkeri* strain MS and *Methanococcus voltae* strain A3) were shown to catalyze the reductive dissolution of FeS_2_ when grown with methanol and acetate or with formate, respectively, when incubated at 38°C, with FeS_2_ as the sole source of Fe and S for cell growth (Payne et al., 2021b). This same study showed that *M. voltae* required direct contact with FeS_2_ to catalyze its reduction and/or to acquire Fe and S dissolution products to meet nutritional demands. The FeS_2_ dissolution product Fe_1–_*_*x*_*S has a relatively high solubility of ∼2 μM (Davison, 1991) at the ionic strength and the circumneutral pH of the base salts medium used to cultivate *M. voltae* in the aforementioned study (Payne et al., 2021b). In the presence of a stoichiometric excess of HS^–^ (>2 μM) and at circumneutral pH, the predominant form of Fe(II) in solution from Fe_1–_*_*x*_*S dissolution is iron monosulfide aqueous (FeS_*aq*_) clusters (Luther and Rickard, 2005; Rickard and Luther, 2007). Given that the measured concentration of HS^–^ significantly exceeded 2 μM (reaching concentrations as high as 35 μM) in the methanogen cultures that were actively shown to be reducing FeS_2_, it was proposed that the cells assimilated soluble FeS_*aq*_ clusters to meet biosynthetic demands (Payne et al., 2021b). These findings are significant since they point to the existence of a biological mechanism that can drive FeS_2_ reductive dissolution and mobilization of Fe and S under anoxic and lower temperature (38°C) conditions that, until recently (Payne et al., 2021b), were thought to stabilize FeS_2_. Further, this newly discovered process may provide an explanation for how methanogens meet their unusually high demand for Fe (Ronnow and Gunnarsson, 1981; Major et al., 2004; Liu et al., 2010, 2012; Johnson et al., 2021) in anoxic and sulfidic habitats, conditions that favor formation of FeS_*aq*_, Fe_1–_*_*x*_*S, and FeS_2_ phases (Luther and Rickard, 2005; Rickard and Luther, 2007). However, the mechanism(s) involved in the reductive dissolution of FeS_2_ by methanogens remains unknown.

Several methanogens have been shown to generate metabolic H_2_ (Valentine et al., 2000; Lupa et al., 2008; Kulkarni et al., 2018), suggesting the possibility that FeS_2_ reduction is indirectly mediated by biogenic H_2_, similar to what has been shown abiotically at high temperature (Hall, 1986; Truche et al., 2010). For example, *M. barkeri* contributes to its proton motive force during metabolism of methanol by producing H_2_ intracellularly and oxidizing it extracellularly (Kulkarni et al., 2018). Likewise, *Methanococcus maripaludis* produces H_2_ during growth with formate through the combined activities of formate dehydrogenase and F_420_-reducing [NiFe]-hydrogenase (Lupa et al., 2008). While the amounts of H_2_ produced by methanogens under such growth conditions are low (∼0.6 mbar, equivalent to ∼0.8 μM aqueous; Kulkarni et al., 2018) and are far lower than the concentrations tested in high temperature abiotic FeS_2_ reduction experiments (>7 mM aqueous; Truche et al., 2010), the possibility exists that H_2_ produced during methanogenic metabolism could mediate FeS_2_ reduction. Possible support for this mechanism comes from the recent discovery of extracellular [NiFe]-hydrogenases mediating extracellular electron transfer (EET) reactions in *M. maripaludis* (Deutzmann et al., 2015).

In the present study, the potential role for H_2_ in the reductive dissolution of FeS_2_ was investigated using a combination of thermodynamic modeling and abiotic experiments. Next, the involvement of biogenic H_2_ in the reduction of FeS_2_ was evaluated in experiments using multiple strains of *M. barkeri* (Fusaro and MS). *M. barkeri* strains were evaluated for H_2_ production during growth with methanol and acetate on FeS_2_ as the sole source of Fe and S. Further, a mutant strain of *M. barkeri* Fusaro with deletions in four operons encoding five [NiFe]-hydrogenases in its genome, rendering it incapable of H_2_ production or consumption (Mand et al., 2018), was used to determine whether biogenic H_2_ and/or [NiFe]-hydrogenases are required for biological FeS_2_ reduction. The necessity for cells to directly contact FeS_2_ to reduce the mineral and/or to assimilate Fe or S from Fe_1–_*_*x*_*S that likely precipitates on the surface of FeS_2_ during reduction was investigated in wild-type *M. barkeri* Fusaro cultures with defined minerals (FeS_2_ or Fe_1–_*_*x*_*S) sequestered in dialysis membranes. Additionally, a model quinone compound, anthraquinone-2,6-disulfonate (AQDS), previously shown to facilitate electron transfer between *M. barkeri* and iron hydroxide minerals (Bond and Lovley, 2002; Liu et al., 2011), was included in cultures provided with FeS_2_ sequestered in dialysis tubing. Lastly, transcriptomic analyses of *M. barkeri* strain MS, which can utilize cysteine or sulfide as the sole S source, were conducted to identify potential proteins or processes involved in FeS_2_ reduction. Collectively, the results from this study are presented in a multistep biogeochemical model to explain how methanogen cells obtain Fe and S from FeS_2_ as FeS_*aq*_ to meet nutritional demands.

## Results and Discussion

### Abiotic Reduction of FeS_2_ by H_2_

Recent studies have shown that methanogens can reductively dissolve FeS_2_ (Payne et al., 2021a,b). Given that (1) methanogens can produce H_2_ during methanogenesis (Lupa et al., 2008; Kulkarni et al., 2018) combined with (2) prior studies that have demonstrated abiotic FeS_2_ reduction by H_2_, albeit at high temperature (>90°C) and high H_2_ partial pressure (>8 bar, equivalent to >7 mM aqueous H_2_; Truche et al., 2010), it was necessary to first evaluate the potential for H_2_ to abiotically reduce FeS_2_ at lower temperature. High temperature (>90°C) abiotic reduction of FeS_2_ occurs according to Eq. 1:


(1) $$FeS_{2}+\left(1-x\right)H_{2}⇆Fe_{1-x}S+\left(1-x\right)HS^{-}+\left(1-x\right)H^{+}$$

where the value of *x* can range from 0 to 0.17 (Hall, 1986; Rickard and Luther, 2007; Truche et al., 2010). The thermodynamics of Eq. 1 were evaluated and abiotic reduction experiments with H_2_ were conducted using laboratory-synthesized nanoparticulate FeS_2_ to determine if abiotic FeS_2_ reduction can occur at temperatures lower than 90°C, to identify the potential products of FeS_2_ dissolution, to quantify the threshold concentration of H_2_ required to generate detectable products of this reaction, and to determine the sensitivity of the reaction to product (i.e., HS^–^) accumulation.

**FIGURE 1:**
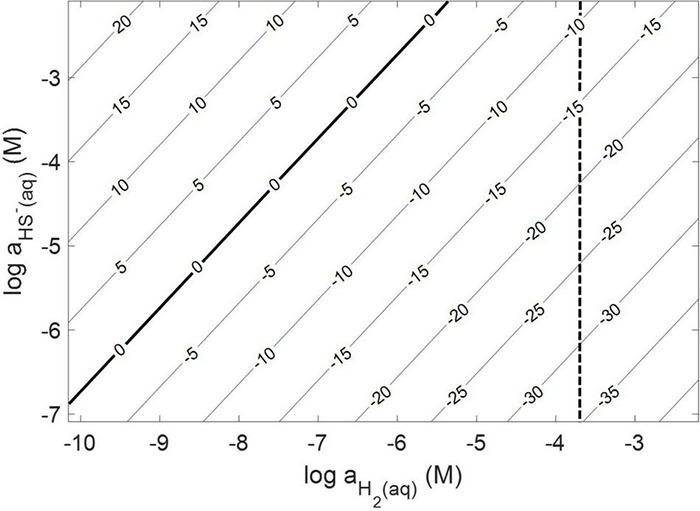
Gibb’s free energy calculated for abiotic pyrite (FeS_2_) reduction to pyrrhotite (Fe_1–_*_*x*_*S) and HS^–^ by H_2_ at 38°C, an ionic strength of 0.05 M, and at a pH of 7.0. The vertical dashed line depicts the minimum H_2_ concentration that abiotic FeS_2_ reduction was detected experimentally (see Figure 2).

Thermodynamic calculations were performed at pH 7.0 and an ionic strength of 0.05 M, a temperature of 38°C, and a free energy of formation for FeS_2_ of –160.2 kJ mol^–1^ (Chareev et al., 2014). These conditions were chosen because they are similar to those used or measured in methanogen cultures demonstrated to reduce FeS_2_ (Payne et al., 2021b). At 38°C, reduction of FeS_2_ by H_2_ was shown to be favorable across a range of aqueous H_2_ concentrations as low as 10^–10^ M and HS^–^ concentrations (10^–7^ to 10^–2^ M), if one assumes that Fe_1–_*_*x*_*S [Fe_0.86_S; free energy of formation of –136 kJ mol^–1^ (Chareev et al., 2014)] is the end product of the reaction (Truche et al., 2010) and using the Davies equation (Stumm and Morgan, 1996) to account for the influence of ionic strength on soluble ion activities (Figure 1). Interestingly, the reaction was not thermodynamically favorable if the end product, Fe_1–_*_*x*_*S, was replaced with the iron monosulfide phase mackinawite [FeS; free energy of formation of –89.2 kJ mol^–1^ (Berner, 1967)] as a product (data not shown). This observation is consistent with the detection of Fe_1–_*_*x*_*S as the primary product of abiotic FeS_2_ reduction, albeit at high temperature [>90°C; (Truche et al., 2010)]. These calculations confirm that abiotic reduction of FeS_2_ to HS^–^ and Fe_1–_*_*x*_*S, but not FeS, by H_2_ is thermodynamically feasible under the conditions of prior experiments (Payne et al., 2021a,b).

**FIGURE 2:**
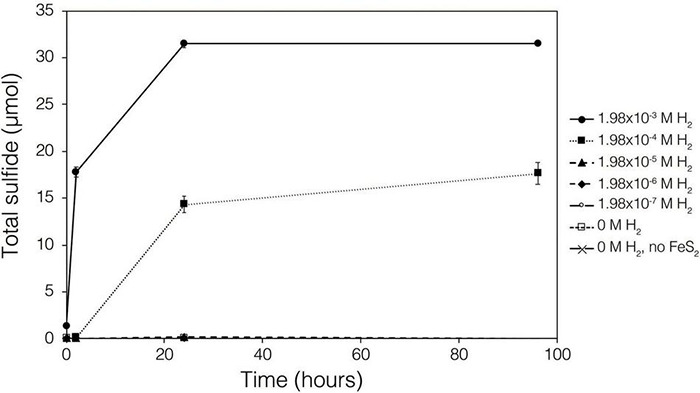
Production of total sulfide (aqueous plus gas phase) in reactors containing 2 mM synthetic FeS_2_ nanoparticles when incubated at 38°C in the presence of H_2_ ranging from 0 to 1.98 × 10^– 3^ M aqueous H_2_ (equivalent to 0 to 2.5 bar). All abiotic reactors contained 35 mL of base salts medium and 35 mL of headspace (balance of headspace as N_2_ gas).

Abiotic experiments were conducted using synthetic FeS_2_ (2 mM in FeS_2_ formula unit) nanoparticles in reactors incubated at 38°C in low salinity, carbonate-buffered medium (2 *g* L^–1^ NaHCO_3_) at pH 7.0, similar to the conditions under which biological FeS_2_ reduction has been demonstrated (Payne et al., 2021a,b) and for which the thermodynamic parameters above were calculated. In reactors with a 100% H_2_ headspace (1.98 × 10^–3^ M aqueous H_2_), rapid abiotic reduction of FeS_2_ was observed, as determined by the production of total sulfide (sum of aqueous HS^–^ and gaseous H_2_S) according to Eq. 1 (Figure 2). Significantly less, albeit still quantifiable, total sulfide was generated in abiotic reactors with a headspace comprising 10% H_2_ (1.98 × 10^–4^ M aqueous). Reactors with headspace H_2_ of 1% (1.98 × 10^–5^ M aqueous) or lower did not yield detectable total sulfide (minimum detection limit = 1.5 μM aqueous concentration) during the 4-day incubation. Nonetheless, this indicates that synthetic FeS_2_ can be reduced at temperatures as low as 38°C, but the products of this reaction are only detectable by colorimetric methods above a threshold H_2_ concentration that exists between 1.98 × 10^–5^ and 1.98 × 10^–4^ M. To confirm that abiotic reduction by H_2_ is not limited to synthetic nanoparticulate FeS_2_, 1.5 g of ground (63–125 μm size fraction) specimen-grade, high-purity FeS_2_ was incubated in 75 mL of base salts medium under 100% H_2_ (1.98 × 10^–3^ M aqueous concentration) or 100% N_2_. After 5 days of incubation at 38°C, significant total sulfide (7.19 ± 0.01 μmol) was generated through abiotic reduction by H_2_ and no sulfide was detected in N_2_ control reactors (data not shown). The concentration of dissolved H_2_ required to reduce FeS_2_ in abiotic experiments was far higher than the minimum (10^–10^ M) estimated by thermodynamic calculations. This suggests that factors other than the free energy for the reduction alone [e.g., particle interfacial energy (Stumm, 1992)] likely play a role in controlling the favorability of the reduction reaction.

In line with previous high-temperature studies of FeS_2_ reduction (Truche et al., 2010), thermodynamic calculations predicted that the favored product of FeS_2_ reduction is Fe_1–_*_*x*_*S. To examine the potential solid phase(s) of Fe(II) and S formed during low temperature FeS_2_ reduction in the presence of H_2_, X-ray diffraction (XRD) analyses of FeS_2_ reacted abiotically under 100% H_2_ headspace for 24 h at 38°C were conducted. The XRD spectra for the H_2_-reacted FeS_2_ was nearly identical to that for the unreacted FeS_2_ (Supplementary Figures 1A,B). Reference peaks for Fe_1–_*_*x*_*S (PDF #97-015-1765) and FeS_*mack*_ (PDF #97-063-3302) were manually searched against the XRD spectra, for both reacted and unreacted FeS_2_, without matches. It is likely that the abundance of Fe_1–_*_*x*_*S, if produced during abiotic H_2_-driven FeS_2_ reduction, remains below the detection limit of XRD (∼10% relative abundance by weight). Additional characterization of FeS_2_ surfaces following reduction by H_2_ using higher resolution spectroscopic methods, such as X-ray absorption spectroscopy, are needed to identify the low abundance secondary mineral(s) that may form on the FeS_2_ surface.

### Dissolution of Fe_1–*x*_S

**FIGURE 3:**
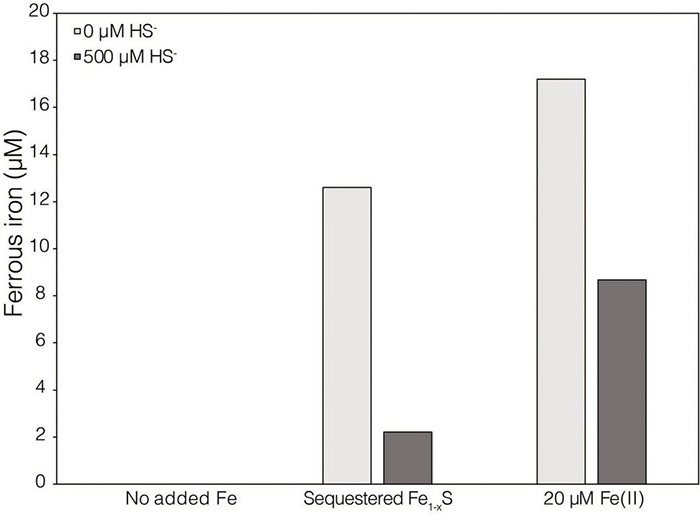
Dissolved ferrous iron [Fe(II)] concentration in abiotic dialysis experiment reactors following 7 days of incubation at 38°C. One hundred sixty-five milliliters serum bottles containing base salts medium were provided with no Fe (No added Fe), 0.1 *g* of specimen pyrrhotite sequestered in 50 kDa dialysis tubing (Sequestered Fe_1–_*_*x*_*S), or 20 μM FeCl_2_. Reactors were either provided with no sulfur source (0 μM HS^–^) or 500 μM Na_2_S (500 μM HS^–^). Following incubation, subsamples were collected under anoxic conditions and were leached with 1N HCl at 4°C for 16 h before quantifying Fe(II).

While XRD analyses did not identify Fe_1–_*_*x*_*S produced during abiotic reduction of FeS_2_ reduction by H_2_, it was still the favored secondary mineral to form on the surface of FeS_2_ based on thermodynamic calculations and observations from studies conducted at higher temperature (Truche et al., 2010). Thus, the solubility of Fe_1–_*_*x*_*S was examined to identify its plausibility as a source of soluble Fe and S. Specimen Fe_1–_*_*x*_*S obtained from Aymar quarry, Gualba mines, Gualba, Montseny, Barcelona Spain, which has previously been shown to be of high purity (de Aldecoa et al., 2013), was used in experiments. XRD of the mineral indicated that the only FeS phase present was Fe_1–_*_*x*_*S (∼80%) with the balance as quartz (Supplementary Figure 1D). Dissolved (<0.2 μm filtered) Fe(II) was detected in abiotic incubations of 0.1 *g* of ground (63–125 μm) Fe_1–_*_*x*_*S following 7 days incubation (Figure 3). While this amount of Fe_1–_*_*x*_*S is greater than what one would expect to form in cultures of *M. barkeri* actively reducing FeS_2_, it does demonstrate that Fe(II) can be solubilized from Fe_1–_*_*x*_*S and is a possible source of Fe used by cells reducing FeS_2_. Interestingly, HS^–^ was not detected (detection limit of 1.5 μM) in solution following this incubation period. This observation is consistent with a previous study that indicated that Fe [as Fe(II)], but not S (as HS^–^), in Fe_1–_*_*x*_*S is mobile and can solubilized from the mineral surface leaving behind a metal-deplete surface layer (Mikhlin, 2000).

Importantly, during FeS_2_ reduction, the total number of moles of HS^–^ formed should be equivalent to the total moles of Fe_1–_*_*x*_*S formed per Eq. 1. Since the dissolution of Fe from Fe_1–_*_*x*_*S is incomplete, the yield of Fe(II) in solution from Fe_1–x_S dissolution is far lower than the yield of total sulfide (>1 μM) during FeS_2_ reduction. Based on thermodynamic data (Luther and Rickard, 2005; Rickard and Luther, 2007), aqueous solutions with HS^–^ in excess of Fe(II) favors hydrated FeS_*aq*_ clusters as the predominant form of Fe(II) in solution. Experiments have shown that the methanogen *M. voltae*, when grown with FeS_2_ as the sole source of Fe and S, simultaneously exhibited evidence indicative of Fe limitation [i.e., up-expression of the Fe(II) transporter FeoB and the metal regulator DtxR] but at the same time hyperaccumulated Fe as a thioferrate-like mineral (Payne et al., 2021a). To explain this paradox, it was suggested that cells incorrectly sensed Fe(II) limitation during growth due to assimilation of Fe(II) complexed with sulfide (i.e., FeS_*aq*_). In this model, excess Fe(II) that was assimilated as hydrated FeS_*aq*_ clusters (cells require more S than Fe) was then sequestered as thioferrate-like nanoparticles to limit toxicity (Payne et al., 2021a). FeS_*aq*_ is predicted to be uncharged at the circumneutral pH of the culture medium used to cultivate *M. voltae* and *M. barkeri* (Jordan et al., 2019a,b). Thus, it is plausible that slightly hydrophobic and uncharged FeS_*aq*_ can either passively diffuse or be actively transported across cellular membranes to provide Fe and S to cells. Additional growth experiments with Fe_1–_*_*x*_*S are described below.

### Involvement of Biogenic H_2_ and [NiFe]-Hydrogenases in Biological Reduction of FeS_2_

**FIGURE 4:**
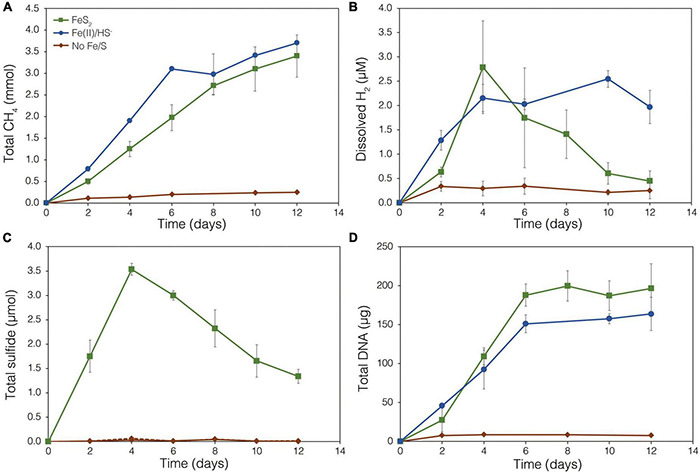
Production of **(A)** total CH_4_, **(B)** dissolved H_2_, **(C)** total sulfide (aqueous plus gas phase), and **(D)** total biomass (DNA) by *Methanosarcina barkeri* strain Fusaro during growth with 2 mM synthetic pyrite (FeS_2_) nanoparticles, 20 μM ferrous iron [Fe(II)] and 2 mM HS^–^, or no added Fe or S source (No Fe/S). The H_2_ concentration is reported for the dissolved phase. Averages and standard deviations for triplicates are shown. Protein data as an additional proxy for growth is presented in Supplementary Figure 3. Data depicting growth kinetics and activities of *M. barkeri* strain MS with FeS_2_ are presented in Supplementary Figure 4.

The demonstration of abiotic reduction of synthetic and specimen FeS_2_ by H_2_, even at low temperature and low H_2_ concentrations, led to an investigation into the potential role of biogenic H_2_ in FeS_2_ reduction by *M. barkeri* strains Fusaro and MS. *M. barkeri* Fusaro was capable of reducing and growing on FeS_2_ as the sole Fe and S source, as indicated by the production of CH_4_, DNA, and total sulfide (Figures 4A,C,D). Cultures of FeS_2_-grown *M. barkeri* Fusaro generated H_2_ at concentrations that were similar to cultures grown with Fe(II) and HS^–^ (Figure 4B). *M. barkeri* strain MS produced slightly more H_2_ during growth on FeS_2_ compared to growth on Fe(II) and cysteine, consistent with increased growth as indicated by higher DNA and CH_4_ production (Supplementary Figure 3B). Regardless, the maximum amount of H_2_ generated by *M. barkeri* strains Fusaro and MS grown with FeS_2_ (2.65 and 2.79 μM aqueous, respectively) was nearly two orders of magnitude below the lowest concentration of H_2_ (198 μM) that was experimentally found to drive abiotic FeS_2_ reduction (Figure 2). While this points to biogenic H_2_ not acting as the mediator of FeS_2_ reduction, it cannot be ruled out that the interface between FeS_2_ minerals and actively growing *M. barkeri* cells could sustain a locally elevated concentration of H_2_ that facilitates indirect, abiotic reduction of the mineral. This is particularly true considering that thermodynamic calculations indicate that far lower concentrations of H_2_ (10^–10^ M aqueous) may be able to drive abiotic FeS_2_ reduction with Fe_1–_*_*x*_*S as the end-product of the reaction (Eq. 1).

**FIGURE 5:**
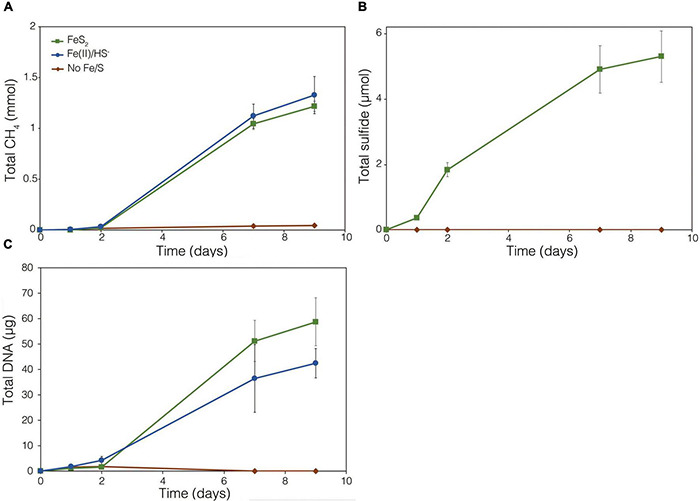
Production of **(A)** methane (CH_4_), **(B)** total sulfide (aqueous plus gas phase), and **(C)** total biomass (DNA) in cultures of *Methanosarcina barkeri* Fusaro with deletions in each of the four operons encoding all five [NiFe]-hydrogenases in its genome. The *M. barkeri* Fusaro hydrogenase mutant was grown either with no provided iron (Fe) or sulfur (S) source (No Fe/S); with 20 μM ferrous iron [Fe(II)] and 0.4 mM sulfide (HS^–^) [Fe(II)/HS^–^]; or with 2 mM synthetic pyrite (FeS_2_) nanoparticles as the sole sources of Fe and S. Hydrogen (H_2_) was not detected (detection limit 0.1 μM) in the headspace of any of the culture conditions tested (data not shown). Averages and standard deviations for triplicates are shown.

To further test whether FeS_2_ reduction is mediated by biogenic H_2_, a strain of *M. barkeri* Fusaro with mutations in all [NiFe]-hydrogenase operons encoded in its genome, rendering the strain unable to consume or produce H_2_ (Mand et al., 2018), was evaluated for its ability to reduce FeS_2_. The strain has mutations in one membrane-associated energy converting [NiFe]-hydrogenase (Ech), two F_420_-reducing [NiFe]-hydrogenases (Frh and Fre), and two membrane-associated methanophenazine-reducing [NiFe]-hydrogenases (Vht and Vhx; Kulkarni et al., 2018). The *M. barkeri* Fusaro [NiFe]-hydrogenase mutant maintained the ability to reduce FeS_2_ as indicated by significant accumulation of HS^–^ in the medium during growth with methanol as the methanogenesis substrate and with FeS_2_ as the sole Fe and S source (Figure 5B). Growth was not detected when a source of Fe or S was not provided (Figures 5A,C). Further, growth of the *M. barkeri* Fusaro [NiFe]-hydrogenase mutant was slightly, albeit significantly (*p* < 0.05), enhanced on FeS_2_ relative to non-mineral sources of Fe and S [Fe(II) and HS^–^] (Figure 5C).

Throughout the growth experiment with the *M. barkeri* Fusaro [NiFe]-hydrogenase mutant, H_2_ remained below the limit of detection (0.1 μM aqueous). This indicates the [NiFe]-hydrogenase mutant strain of *M. barkeri* Fusaro did not produce H_2_, yet it maintained the ability to reduce FeS_2_ and assimilate dissolution products to meet Fe and S biosynthetic demands. It follows that indirect reduction of FeS_2_ through biogenic H_2_ is not the mechanism of biological FeS_2_ reduction. Further, these results indicate that [NiFe]-hydrogenases (Ech, Frh, Fre, Vht, and/or Vhx) themselves are not involved in FeS_2_ reduction, ruling out H_2_ or [NiFe]-hydrogenase mediated EET as a mechanism to drive FeS_2_ reduction. This is potentially consistent with previous results suggesting that H_2_ and [NiFe]-hydrogenases do not play a role in EET from the cells to external electron acceptors in *M. barkeri* strain Fusaro (Rowe et al., 2019). However, the directionality of EET from *M. barkeri* cells to FeS_2_ differs from that of the previous study which focused on electron acquisition from cathodes to *M. barkeri* Fusaro cells *via* EET (Rowe et al., 2019). Further, while electron acquisition from cathodes *via* EET was apparently respiratory in nature (Rowe et al., 2019), the reduction of FeS_2_ as described herein fulfills the purpose of generating bioavailable forms of Fe and S to meet nutritional demands. It is thus possible that the mechanisms of EET for *M. barkeri* in the growth conditions described herein, versus those described previously, differ.

### *Methanosarcina barkeri* Requires Direct Contact to Reduce FeS_2_ but Not to Assimilate FeS_2_ Reduction Products

Prior work showed that *M. voltae* A3 could not grow or reduce synthetic FeS_2_ when physical access to the mineral was restricted using dialysis tubing (Payne et al., 2021b). However, it remained unclear whether cells required direct access to the mineral to (1) carryout reduction and/or to (2) acquire Fe and S from the Fe_1–_*_*x*_*S that is predicted to precipitate on the surface of FeS_2_ following reduction. Field emission microscopy (FEM) was used to visualize physical associations between *M. barkeri* Fusaro cells and minerals during growth with FeS_2_ as the sole source of Fe and S. FEM shows that *M. barkeri* cells directly associated with FeS_2_ surfaces during growth (Supplementary Figure 5). *M. barkeri* is known to associate with other solid phases, such as cathodes, *via* extracellular polymeric substances (EPS; Rowe et al., 2019), thus a similar mechanism may be employed to attach to FeS_2_.

**FIGURE 6:**
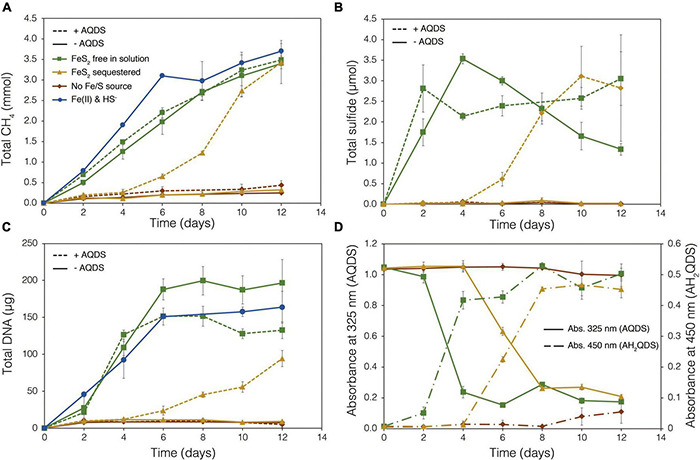
Production of **(A)** total methane (CH_4_), **(B)** total sulfide (aqueous plus gas phase), **(C)** total biomass (DNA), and **(D)** reduced anthrahydroquinone-2,6-disulfonate (AH_2_QDS) from anthraquinone-2,6-disulfonate (AQDS) by *Methanosarcina barkeri* strain Fusaro when grown with pyrite (FeS_2_) free in solution (green) or sequestered in 100 kDa dialysis tubing (orange) to prevent physical association. Negative control cultures contained no added Fe or S source (red), while positive control cultures were provided with 20 μM Fe(II) and 2 mM HS^–^ (red; same data as presented in Figure 2). AQDS was provided at a final concentration of 20 mM. In **(A–C)**, culture conditions with added AQDS are represented by dashed lines and culture conditions that do not contain AQDS are shown by solid lines. Only conditions provided AQDS are shown panel **d**, and absorbance at 325 nm (AQDS) is depicted by solid lines while absorbance at 450 nm (AH_2_QDS) depicted by long-dashed lines. Averages and standard deviations for triplicates are shown. Protein data as an additional proxy for growth is presented in Supplementary Figure 3.

To examine the requirement for *M. barkeri* Fusaro cells to directly contact FeS_2_ in order to grow, synthetic FeS_2_ was sequestered in dialysis tubing with 100 kDa diameter pore size to prohibit physical contact of the cells with the mineral surface. This also excluded extracellular organic or inorganic molecular complexes with diameters larger than 100 kDa from interacting with the mineral surface. Under a subset of conditions, the synthetic quinone, AQDS, previously shown to act as an electron shuttle between *M. barkeri* and Fe-oxide minerals (Bond and Lovley, 2002), was provided to cells grown with FeS_2_ in solution and sequestered in dialysis tubing. This allowed for the determination of whether (1) direct electron transfer from the methanogen cell surface was required to reduce the mineral, (2) AQDS could enable FeS_2_ reduction by acting as an electron shuttle between the cell and FeS_2_, and/or (3) the products of FeS_2_ reduction could pass through the dialysis membrane to support growth. Cells that were provided with direct access to FeS_2_ rapidly reduced the mineral as indicated by total sulfide accumulation up to 3.53 μmol (30.5 μM aqueous sulfide) before the concentration slowly started to decrease, presumably due to its ultimate utilization by the cells (Figure 6B). Concurrently, cells generated CH_4_ and biomass, as determined by DNA production (Figures 6A,C) and protein production (Supplementary Figure 3). When FeS_2_ was sequestered in dialysis membranes and no AQDS was provided, *M. barkeri* Fusaro could not reduce FeS_2_ as indicated by the lack of sulfide accumulation in the growth medium (Figure 6B). Furthermore, production of CH_4_ and biomass (DNA and protein) were 1–2 orders of magnitude less when access to FeS_2_ was restricted by sequestering FeS_2_ in dialysis tubing when compared to when FeS_2_ was not sequestered and were not significantly different from controls where no Fe or S were provided (Figure 6A).

Methanogens can transfer electrons to AQDS, generating the reduced form anthrahydroquinone-2,6-disulfonate (AH_2_QDS). The conversion of AQDS to AH_2_QDS was monitored spectrophotometrically by observing a shift in absorbance from 325 to 450 nm (Bond and Lovley, 2002) in cultures provided with direct access to FeS_2_ or when physical contact between cells and FeS_2_ was prevented using dialysis tubing. *M. barkeri* rapidly reduced AQDS to AH_2_QDS in cultures with FeS_2_ free in solution (Figure 6D), and this corresponded to accelerated HS^–^ production from FeS_2_ reduction and enhanced cell growth and CH_4_ production activity (Figures 6A–C). Furthermore, the concentration of HS^–^ remained steady during growth and even increased slightly toward the end of the methanogen growth phase, suggesting that cells continued to transfer electrons to AQDS, which in turn continued to reduce FeS_2_ even after cell growth had ceased. When FeS_2_ was sequestered in dialysis tubing, the conversion of AQDS to AH_2_QDS by *M. barkeri* lagged by approximately 2–4 days. However, following this lag phase, AQDS was reduced, and production of sulfide and cells occurred (Figures 6B,C). Despite the lag in activity and growth, the addition of AQDS as a soluble electron shuttle enabled comparable CH_4_ and biomass (DNA and protein) production in cultures where physical contact between cells and FeS_2_ was limited to those provided with FeS_2_ free in solution. This indicated that *M. barkeri* requires physical contact with FeS_2_ to directly transfer electrons to the mineral surface invoking EET as the primary mechanism for FeS_2_ reduction. However, soluble electron shuttles such as AQDS (a humic acid analog) can allow for mineral reduction when direct contact between cells and minerals is restricted. Further, this indicates that *M. barkeri* does not produce endogenous soluble electron shuttles capable of FeS_2_ reduction but may take advantage of exogenous shuttles (e.g., humic acids, quinones) in natural systems if they are available.

**FIGURE 7:**
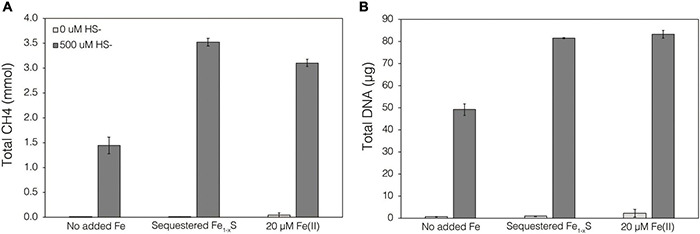
Production of **(A)** total CH_4_ and **(B)** total biomass (DNA) of *M. barkeri* Fusaro wild-type grown with no added Fe (No added Fe), with 0.1 *g* specimen pyrrhotite (Fe_1–_*_*x*_*S; 63–125 μm size fraction) sequestered in 100 kDa dialysis tubing (Sequestered Fe_1–_*_*x*_*S), or with 20 μM FeCl_2_. For each of these conditions, no sulfide (0 μM HS^–^) or 500 μM sulfide (500 μM HS^–^) was added as Na_2_S. The data represent the difference for each analyte between the initial measurement (day 0) and the final measurement (day 10). Averages and standard deviations for triplicates are shown. Sampling was infrequent to minimize potential damage to dialysis membranes from sharp, freshly fractured, Fe_1–_*_*x*_*S grains and their edges.

Thermodynamics experiments described above predicted that the byproducts of FeS_2_ reduction are HS^–^ and a secondary mineral, Fe_1–_*_*x*_*S, which is consistent with previous observations of abiotic FeS_2_ reduction products at high temperature (Truche et al., 2010). To evaluate if *M. barkeri* Fusaro can use Fe_1–_*_*x*_*S as a source of Fe and/or S for growth and to determine if direct contact with this mineral is required for its acquisition and assimilation, dialysis experiments were conducted using ground (63–125 μm) specimen grade Fe_1–_*_*x*_*S particles as the sole Fe source. Ground Fe_1–_*_*x*_*S particles were sequestered in 100 kDa dialysis tubing and reactors were then amended with either 0 or 500 μM HS^–^ as a S source, since abiotic dissolution of Fe_1–_*_*x*_*S was shown to release Fe(II) but not HS^–^, as discussed above. When 0 μM HS^–^ was provided, no CH_4_ production or growth was detected in any condition (Figure 7). However, when cultures were provided with 500 μM HS^–^, activity and growth with sequestered Fe_1–_*_*x*_*S was comparable to growth with 20 μM Fe(II) and 500 μM HS^–^ (Figure 7). Cultures provided with no Fe and 500 μM HS^–^ demonstrated activity and growth, albeit CH_4_ production was 53–59% lower and biomass 40–41% lower than in cultures grown with 20 μM Fe(II) or provided with Fe_1–_*_*x*_*S sequestered in dialysis tubing, respectively. This apparent growth under Fe-limiting conditions is comparable to what has been reported previously for methanogens and is attributed to trace Fe contamination in reagents, in particular Na_2_S, despite its American Chemical Society (ACS) grade (Payne et al., 2021b). Collectively, these data indicate that cells require direct access to the surface of FeS_2_ to reduce the mineral but not to acquire dissolution products, as the solubility of Fe_1–_*_*x*_*S with respect to Fe(II) can support growth of *M. barkeri*. Further, these observations indicate that the source of S for growth is HS^–^ from the initial FeS_2_ reduction step, not Fe_1–_*_*x*_*S. As stated above, the predominant form of Fe(II) solubilized from Fe_1–_*_*x*_*S in sulfidic solutions would be as FeS_*aq*_, which is readily produced in aqueous solutions containing excess HS^–^ relative to Fe(II), making this the likely form of Fe and S that is assimilated.

### Transcriptional and Genomic Insights Into Cell Growth *via* Biological FeS_2_ Reduction

Because FeS_2_ is insoluble in water (equilibrium solubility at 25°C, 0.05 M ionic strength, and pH 7 = ca. 0.1 μM), as summarized by Rickard and Luther (2007), the reduction of persulfides in FeS_2_ by methanogens must occur extracellularly through EET mediated by large (>100 kDa) redox-active molecules or direct electron transfer (e.g., involving electronic conduits within pili, membrane-bound oxidoreductases, secreted molecules, etc.). Genes encoding proteins involved in reducing the persulfide in FeS_2_ through EET might be expected to be upregulated during growth on FeS_2_ as the sole source of Fe and S relative to growth soluble Fe and S sources. Shotgun transcriptomics data collected from *M. barkeri* strain MS were examined to identify protein-encoding genes whose expression was differentially regulated during growth on FeS_2_ (Supplementary Figures 4A–D). Strain MS was selected as it can use cysteine as its sole sulfur source in addition to HS^–^. This provides a suitable control for examining possible deleterious effects of minimal HS^–^ generated from FeS_2_ reduction on growth relative to growth in the presence of 2 mM Na_2_S [Fe(II)/HS^–^], an important consideration given reports of HS^–^ toxicity to cells (Edgcomb et al., 2004). *M. barkeri* strain MS encodes the same complement of [NiFe]-hydrogenases as the wild-type *M. barkeri* strain Fusaro (Mand et al., 2018; Mand and Metcalf, 2019). Consistent with the results described above indicating that H_2_ and [NiFe]-hydrogenases are not involved in FeS_2_ reduction, none of the five [NiFe]-hydrogenases were differentially expressed during growth on FeS_2_ compared to growth on Fe(II)/Cys or Fe(II)/HS^–^ (Supplementary Figure 4E).

**FIGURE 8:**
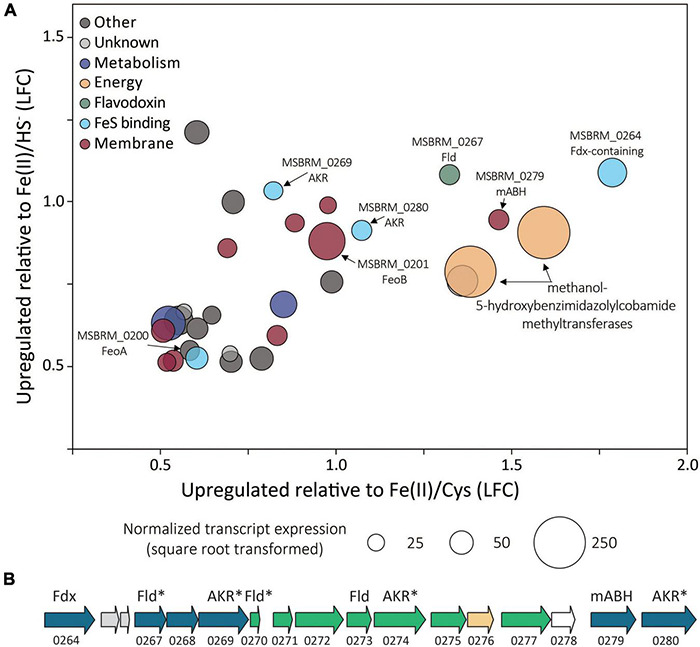
**(A)** *Methanosarcina barkeri* MS gene transcripts that were significantly upregulated in cells provided with synthetic pyrite (FeS_2_) nanoparticles compared to those provided with ferrous iron [Fe(II)]/cysteine (Cys) and those provided with Fe(II)/sulfide (HS^–^) as the sole iron and sulfur source. The log fold change (LFC) of transcript abundance in FeS_2_- versus Fe(II)/HS^–^-grown cells (*y*-axis) is plotted as a function of the LFC of transcript abundance in FeS_2_- versus Fe(II)/Cys-grown cells (*x*-axis). Each point represents a single gene and the size of the point represents the relative mean normalized transcript abundance detected across FeS_2_ replicate cultures. **(B)** A cassette of genes whose expression (transcripts) were upregulated when *Methanosarcina barkeri* MS cells were provided with synthetic FeS_2_ nanoparticles when compared to cells provided with 20 μM ferrous iron [Fe(II)] and 2 mM Cys or 20 μM ferrous iron [Fe(II)] and 2 mM HS^–^ as sole sources of Fe and S. Truncated locus tags and annotations of gene functions as assessed by UniProt are provided (“*MSBRM*_” has been removed due to space limitations). Gene arrows colored blue represent significant differential expression in *M. barkeri* MS cells grown on FeS_2_ compared to cells grown with Fe(II)/Cys or Fe(II)/HS^–^ (*p* < 0.05, LFC > 0.5). The green gene arrows were significantly upregulated (*p* < 0.05) in the FeS_2_ growth condition relative to both the Fe(II)/Cys or Fe(II)/HS^–^ growth conditions but the LFC was below 0.5. The gene depicted in yellow was upregulated on the FeS_2_ growth condition relative to both the Fe(II)/Cys or Fe(II)/HS^–^ growth conditions but not significantly (*p* > 0.05). The gene depicted in white was not differentially regulated among growth conditions. Importantly, an analysis of proteomes of *Methanococcus voltae* A3 cells provided with 2 mM FeS_2_ or 26 μM Fe(II) and 2 mM HS^–^ independently identified homologs of many of the same proteins as being up-expressed under the FeS_2_ condition (Payne et al., 2021a), and these are indicated with an asterisk (*). Gene abbreviations: Fld, flavodoxin; AKR, aldo/keto reductase; Fdx, ferrodoxin; mABH, membrane-associated alpha/beta hydrolase; FeoAB, ferrous iron transporter subunits **(A,B)**.

Of the 3,413 genes covered in the differential transcriptomics experiment, transcripts of only 30 were significantly upregulated [*p* value < 0.05, log_2_ fold change (LFC) > 0.5] and met abundance thresholds (>0.005% of total normalized read count) when cells were grown on FeS_2_ relative to those grown with either Fe(II)/cysteine or Fe(II)/HS^–^ (Figure 8A and Supplementary Table 1). This collection of genes included those that code for 12 hypothetical proteins and seven proteins annotated as being involved in translation, ribosomal processing, and other growth-related functions. Nine of the identified FeS_2_-upregulated genes coded for proteins that are predicted to be membrane-associated. This included genes encoding ABC transporters of organic compounds (*MSBRM*_0980, *MSBRM*_0837, and *MSBRM*_2090) and ferrous iron in its hexaquo Fe(II) form (FeoB; *MSBRM*_0201; Lau et al., 2016). A gene coding for the transcriptional regulator for FeoB, *feoA* (*MSBRM*_0200; Lau et al., 2016), was also identified in this subset of genes upregulated during growth on FeS_2_. A model to rationalize the upregulation of transcripts of FeoAB when FeS_2_ is provided as the sole Fe source versus Fe(II) was outlined in a recently published paper that focused on *M. voltae* A3 (Payne et al., 2021a). Briefly, and as stated above, FeS_2_-grown *M. voltae* cells (and possibly *M. barkeri* cells) were proposed to transport Fe(II) that is complexed with sulfide (FeS_*aq*_; Truche et al., 2010). It was hypothesized that assimilation of Fe(II) as FeS_*aq*_ led cells to sense limited availability of free/dissociable Fe(II) inside the cell *via* Fe(II)-binding transcription factors (i.e., DtxR, FeoA). As such, cells upregulated the hexaquo Fe(II) transporter (FeoB), and to a lesser extent, its transcriptional regulator (FeoA) and global metal regulator (DtxR), to overcome perceived Fe(II) limitation. Evidence indicated that excess transported Fe (cells require more S than Fe) was sequestered intracellularly as thioferrate-like molecules (Payne et al., 2021a). To this end, FeoAB are not hypothesized to be directly involved in FeS_2_ reduction, FeS_*aq*_ assimilation, or in supplying reducing equivalents for EET reactions involving FeS_2_.

A cluster of differentially expressed genes that are co-localized on the *M. barkeri* MS genome that included several oxidoreductases was identified (Figure 8B). Among the 15 genes that comprise this cluster, six were significantly upregulated (*p* value < 0.05, LFC > 0.5) during growth on FeS_2_. An additional eight genes were moderately upregulated with a LFC below 0.5, and one additional gene was upregulated though its expression was not significantly different from Fe(II)/HS^–^- or Fe(II)/cysteine-grown cells. This cluster of genes codes for several enzymes predicted to be cytoplasmic, including one ferredoxin-domain containing oxidoreductase (Fdx; *MSBRM*_0264), three paralogous flavodoxin domain-containing oxidoreductases (Fld; *MSBRM*_0267, *MSBRM*_0270, *MSBRM*_0273), and three paralogous aldo-keto reductases (AKR; *MSBRM*_0269, *MSBRM*_0274 and *MSBRM*_0280). Two proteins were predicted to be membrane-associated including a putative alpha-beta hydrolase (mABH; *MSBRM*_0279) and an additional membrane-associated protein of unknown function (*MSBRM*_0268). Furthermore, a recently published shotgun proteomics study of *M. voltae* A3 cells grown with 2 mM FeS_2_ as the sole Fe and S source compared to cells grown with 26 μM Fe(II) and 2 mM HS^–^ revealed significant up-expression (*p* < 0.05, LFC > 1) of two homologs of Fld (*Mvol*_0029, *Mvol*_0089) and three homologs of AKR (*Mvol*_0066, *Mvol*_0126, and *Mvol*_0416) in FeS_2_-grown cells (Payne et al., 2021a), all of which were predicted to be cytoplasmic. Homologs of mABH and the protein of unknown function (*MSBRM*_0268) were not upregulated in FeS_2_-grown *M. voltae* cells. The Fld and AKR homologs are not co-localized in the genome of *M. voltae* A3 although the majority are co-localized in the genome of *M. barkeri* Fusaro. Yet, the similarities in their regulation and expression in *M. barkeri* MS and *M. voltae* A3 and their cytoplasmic cellular location point to the potential role of these putatively redox-active enzymes in FeS_2_-dependent growth, as discussed in detail below.

To date, five strains representing four methanogen species, *M. voltae* A3 (Payne et al., 2021b), *M. barkeri* MS (Payne et al., 2021b), *M. barkeri* Fusaro (this work), an unnamed *Methanothermobacter* strain isolated from a New Zealand hot spring (provided by Matthew Stott, unpublished data), and *M. maripaludis* S2 (unpublished data) have been shown to reduce FeS_2_ to meet biosynthetic demands for Fe and S. These four species represent evolutionarily and ecologically distinct lineages of methanogens, including more deeply diverging members (*M. voltae* A3, *M. maripaludis* S2, uncharacterized *Methanothermobacter*) and more recently diverging members (*M. barkeri* MS, *M. barkeri* Fusaro; Bapteste et al., 2005; Brochier-Armanet et al., 2011; Petitjean et al., 2015). This suggests that the ability of methanogens to reduce FeS_2_ may be widespread among this group of microorganisms. As such, homologs of the 30 genes upregulated on FeS_2_ relative to Fe(II)/cysteine and Fe(II)/HS^–^ were surveyed in the genomes of four of the five known FeS_2_ reducers (a genome is not available for the uncharacterized *Methanothermobacter* strain).

Homologs for 19 of the 30 upregulated FeS_2_ genes were detected in all four FeS_2_-reducing methanogens with available genomes (Supplementary Table 1). Five of these 19 genes encoded proteins that were related to ribosomal function, five were related to membrane transport, and two were related to energy or metabolism (Gene IDs provided in Supplementary Table 1). Five of the remaining 19 conserved genes were predicted to encode oxidoreductases (Fdx, Fld, and two AKR) and one of the genes is predicted to encode the hydrolase, mABH (Figure 8). Collectively, these data suggest that one or more proteins encoded in this gene cluster (i.e., Fdx, Fld, AKR, and/or mABH) may be involved in a function associated with FeS_2_ reduction as they are conserved across methanogens capable of this functionality.

### Mechanism of Extracellular Electron Transport Involved in FeS_2_ Reduction

Several dedicated mechanisms for EET have been proposed to explain the ability of microorganisms to transfer electrons to minerals, cathodes, or other microorganisms (Shi et al., 2016; Jorgensen, 2021; Rotaru et al., 2021). Notably, all the examples of EET to date have been described for their role in cellular respiration. However, EET to FeS_2_ by methanogens for mineral reduction is likely related to nutrient acquisition as FeS_2_ can serve as the sole source of Fe and S.

Methanogens, including those from the *Methanosarcina* genus, are capable of Fe(III) oxide mineral reduction as a means for energy conservation (Bond and Lovley, 2002; Sivan et al., 2016; Shang et al., 2020). It has been suggested that the mechanism of Fe(III)-oxide reduction mimics that for bacterial species, whereby *Methanosarcina acetivorans* cells use transmembrane, multiheme *c*-type cytochromes (MHC) encoded by *mmcA* to transfer electrons extracellularly to Fe(III) oxides (Holmes et al., 2019), thereby diminishing the rate of methanogenesis, possibly due to diversion of electrons away from methanogenic pathways. However, neither of the genomes of the *M. barkeri* strains used in this study (Fusaro or MS) or the genome of *M. voltae* A3 (Payne et al., 2021b) encode homologs of MHC (data not shown). Further, it has recently been shown that while MHC have been implicated in direct interspecies electron transfer between syntrophic *Geobacter* and *Methanosarcinales*, these molecular complexes are not actually required (Yee and Rotaru, 2020). The possibility exists for indirect EET *via* electron shuttling molecules such as flavins or enzymes such as [NiFe]-hydrogenase and formate dehydrogenase that are excreted from methanogen cells that then drive FeS_2_ reduction. Indeed, *M. maripaludis* has been shown to excrete extracellular enzymes, including both [NiFe]-hydrogenases and formate dehydrogenases, that are capable of driving corrosion of metallic iron (Deutzmann et al., 2015). However, in the present study it was shown that when *M. barkeri* was grown in medium with FeS_2_ sequestered in dialysis tubing with a pore size of 100 kDa, cells were unable to reduce FeS_2_, as evinced by the lack of HS^–^ production and growth (Figure 5). This was also observed for *M. voltae* grown with formate as methanogenesis substrate (Payne et al., 2021b). Thus, a secreted electron shuttling molecule or enzyme would need to have a hydrodynamic diameter large enough to be blocked by the 100 kDa pore, which excludes involvement of low-molecular-weight endogenously produced electron shuttling molecules like flavins. Growth studies with the [NiFe]-hydrogenase *M. barkeri* Fusaro mutant rule out involvement of hydrogenase enzymes in FeS_2_ reduction. Nevertheless, at this time, enzymes and enzyme complexes with large molecular weights (>100 kDa) cannot necessarily be ruled out as being involved in FeS_2_ reduction.

A recent study showed that *M. barkeri* is capable of assimilating and/or precipitating nanoparticulate magnetite intracellularly, and these conductive nanoparticles enhanced methanogenesis activity presumably by acting as solid electron shuttles across the cell membrane (Fu et al., 2019). Given the production of FeS phases (e.g., Fe_1–_*_*x*_*S and/or FeS) during FeS_2_ reduction (Eq. 1), it seems plausible that they may participate in EET in methanogens in a manner similar to that of *Shewanella* (Kondo et al., 2015). Follow-up studies using high-resolution imaging and spectroscopic approaches combined with electrochemical approaches will advance understanding of the likely role of FeS nanoparticles in EET and FeS_2_ reduction.

Without a clear mechanism to describe biological FeS_2_ reduction emerging from experimental data, a model for spontaneous electron transfer from the cell surface to FeS_2_ is considered. In this model, reduction of FeS_2_ may occur by diversion of low potential electrons from the membrane, extracellular proteins, or other components of the extracellular milieu. While it is not yet clear what membrane and extracellular components are involved in FeS_2_ reduction, experiments that showed both AH_2_QDS and H_2_ can abiotically reduce FeS_2_ allow for an estimate of the reduction potential of electrons involved in mineral reduction.

The calculated non-standard state electrochemical potential (*E*) of H_2_ [*E*°= –414 mV (Thauer et al., 1977)] at a headspace partial pressure of 0.25 bar (198 μM dissolved H_2_) in pH 7.0 medium at 38°C is –318 mV, indicating that electrons under such conditions would need to have a midpoint potential equal to or lower than this to reduce FeS_2_. However, thermodynamic calculations conducted herein indicate that FeS_2_ reduction is favorable at H_2_ concentrations as low as 1 μbar (10^–10^ M), which equates to electrons with midpoint potentials of –123 mV. Furthermore, the standard state reduction potential of AH_2_QDS has been estimated to be –184 mV (Clark, 1960), which is lower than that for H_2_ at a concentration of 10^–10^ M, consistent with presented evidence indicating it can facilitate EET to FeS_2_ (Figure 6). Numerous membrane-associated oxidoreductases in methanogens are involved in redox reactions involving electrons that are far more reduced than –184 mV (Thauer et al., 1977), suggesting that their diversion *via* an undefined or even undedicated mechanism toward FeS_2_ is feasible. Alternatively, small redox-active molecules produced by methanogens, such as methanophenazine [*E* = –165 mV (Beifuss and Tietze, 2005)] may be involved in shuttling electrons across the cell membrane to the FeS_2_ surface. This would imply, however, that the mechanism involved in biological FeS_2_ reduction is not universal across FeS_2_-reducing methanogens since *M. voltae* does not produce methanophenazine (Deppenmeier et al., 1999).

## Conclusion

Abiotic reduction of specimen and synthetic, nanoparticulate FeS_2_ by H_2_ at concentrations as low as 1.98 × 10^–4^ M was demonstrated herein. While the initial reduction reaction of FeS_2_ is necessary to generate HS^–^, the solubility of the likely FeS_2_ reduction product, Fe_1–_*_*x*_*S, controls the availability of Fe(II). Production of excess sulfide (>1 μM) *via* FeS_2_ reduction favors formation of FeS_*aq*_ as the dominant form of Fe(II) in solution, which is hypothesized to be the form of Fe and S assimilated by FeS_2_-reducing methanogen cells. Dialysis experiments indicate that *M. barkeri* requires direct contact to reduce FeS_2_ through EET but not to obtain dissolution products to meet nutrient demands. Growth experiments with wild-type and mutant strains of *M. barkeri* indicate that H_2_ and [NiFe]-hydrogenase are not involved in EET and that the cells do not naturally produce electron shuttles capable of reducing EET. Synthetic electron shuttles such as AQDS can facilitate EET to FeS_2_, suggesting exogenous compounds with similar reduction potentials (quinones, humic acids) could serve this role in natural systems. Transcriptomic experiments did not identify differential expression of genes putatively involved in EET, although a cassette of genes that encodes several oxidoreductases, including Fdx, Fld, and AKRs, and mABH, was found to be significantly upregulated during growth on FeS_2_ relative to Fe(II)/cysteine and Fe(II)/HS^–^. It is possible that these proteins are involved in either supplying reducing equivalents for EET to FeS_2_ or involved in processing, trafficking, storing, or transforming FeS_*aq*_ that is likely assimilated as the sole source of Fe and S during growth with FeS_2_ reduction. As such, the mechanism(s) of EET from methanogen cells to FeS_2_ requires direct contact between cells and the mineral and may involve electrically conductive components of EPS or electrically conductive FeS nanoparticles that self-assemble into conductive conduits within EPS that interfaces cells and the mineral surface.

**FIGURE 9:**
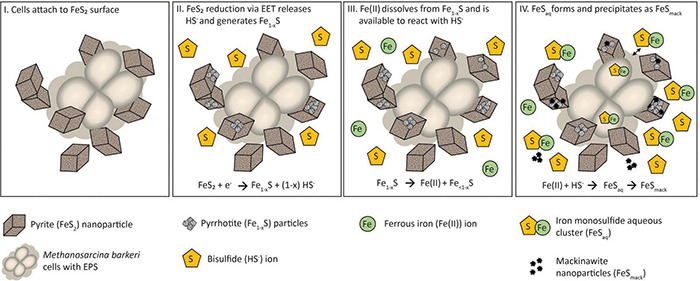
A model for *Methanosarcina barkeri* growth on pyrite (FeS_2_). See main text for description.

A multi-step model is proposed to describe the reduction of FeS_2_ and the assimilation of Fe and S during growth with FeS_2_ and to guide future experiments (Figure 9). Cells first attach to the FeS_2_ surface, allowing for microbial reduction of FeS_2_ *via* a cell surface mediated mechanism of EET (Step 1). During FeS_2_ reduction, soluble HS^–^ is generated and Fe_1–_*_*x*_*S precipitates on the surface of FeS_2_ (Step 2). Dissolution of Fe(II; but not S) from Fe_1–_*_*x*_*S occurs, and because dissolution is incomplete, the concentration of HS^–^ in solution exceeds that of Fe(II; Step 3). This favors the formation of FeS_*aq*_ clusters as the primary form of Fe(II) in solution (Step 4). FeS_*aq*_ clusters may passively diffuse or be actively transported across the membrane to meet Fe and S nutritional demands. FeS_*aq*_ clusters, which themselves are likely to be conductive, may also associate with the surface of the cell, EPS, or mineral, thereby enhancing EET to FeS_2_ (Step 4). This model clarifies the surface-requirement of methanogens during growth on FeS_2_ to that of EET, rather than for Fe or S acquisition from Fe_1–_*_*x*_*S. Further, it highlights the complexities methanogens face in obtaining Fe and S to meet nutritional demands in anoxic environments where FeS, Fe_1–_*_*x*_*S, and FeS_2_ are likely to be prevalent forms of these elements (Rickard and Luther, 2007).

## Materials and Methods

### Mineral Preparation

Synthetic nanoparticulate pyrite (FeS_2_) was prepared as previously described (Payne et al., 2021b) and XRD analysis is provided in Supplementary Figure 1. ACS grade chemicals were used in mineral syntheses (and preparation of cultivation medium, described below) and glassware was washed with 10% trace-metal grade nitric acid (HNO_3_) to remove potential trace metal impurities. Briefly, the initial FeS_2_ synthesis reaction was conducted in an anaerobic chamber (2.5% H_2_/balance N_2_) and was then bubbled with sterile N_2_ gas passed over a heated (200°C) and H_2_-reduced column containing native copper shavings for 1 h L^–1^. Following bubbling, FeS_2_ was incubated anoxically (N_2_ headspace) in a sealed, serum bottle for 4 days at 65°C followed by an additional 4 days at 85°C to complete the synthesis. Finally, to remove unreacted HS^–^, Fe(II), FeS, and S^0^, the synthesized nanoparticulate FeS_2_ was washed (via centrifugation and decanting) four times with 1N HCl, once with boiling 6N HCl, twice with MilliQ (MQ) H_2_O, three times with >99.5% acetone, and finally three times with sterile, anoxic MQ H_2_O inside of an anaerobic chamber. Following the final wash step, the FeS_2_ was resuspended in sterile, anoxic MQ H_2_O and transferred to a sterile serum bottle that was then capped and sealed prior to removal from the anaerobic chamber. Upon removal from the anaerobic chamber, the headspace of the serum bottle was flushed with 0.22 μm filtered N_2_ gas. To determine the concentration of the synthesized FeS_2_ slurry, triplicate 1 mL aliquots were transferred to pre-weighed serum bottles, dried anoxically under N_2_ gas, and weighed to calculate the weight percent and molar concentration. The concentration of the FeS_2_ stock was adjusted to 0.2 M by diluting into sterile, anoxic MQ H_2_O.

Specimen grade FeS_2_ was obtained from Zacatecas, Mexico (Ward’s Science, Rochester, NY, United States) and specimen grade Fe_1–_*_*x*_*S was obtained from Aymar quarry, Gualba mines, Gualba, Montseny, Barcelona Spain. Within a laminar flow hood, specimen minerals were crushed with a MQ-cleaned and ethanol-sterilized jaw crusher (Gilson, Lewis Center, OH, United States) then applied to a cleaned and sterile sieve stack (United States Standard #10/2,000 μm, United States Standard #35/500 μm, United States Standard #60/250 μm, United States Standard #120/125 μm, United States Standard #230/63 μm, and catch pan; all 8” diameter). The 63–125 μm fractions were collected and washed. Specimen FeS_2_ was washed as described above for synthetic FeS_2_ while specimen Fe_1–_*_*x*_*S was washed three times with >99.5% acetone and then three times with sterile, anoxic MQ within an anaerobic chamber. The washed minerals were transferred to sterilized, N_2_ purged, sealed serum bottles then dried under a stream of 0.22 μm filtered N_2_ gas. The dried minerals were characterized using a SCINTAG X-1 system X-ray powder diffraction (XRD) spectrometer (XRD Eigenmann GmbH, Mannheim, Germany).

### Abiotic FeS_2_ Reduction Experiments

Synthetic, nanoparticulate FeS_2_ (2 mM) was added to triplicate reactors that contained 35 mL of low salinity basal medium (in *g* L^–1^: NaCl, 1.00; MgCl_2_ ⋅ 6H_2_O, 0.40; NH_4_Cl, 0.50; KCl, 0.50; CaCl_2_ ⋅ 2H_2_O, 0.10; KH_2_PO_4_, 0.15_;_ NaHCO_3_, 2.0) in 70 mL glass serum bottles capped with blue butyl rubber stoppers (Bellco, Vineland, NJ, United States). This medium is similar to growth medium for *M. barkeri* MS described below, but without methanol, acetate, vitamins, or trace metal solutions. The reactors were then purged for 20 min with 0.2 μm filtered N_2_ that had been passed over a heated (200°C) and H_2_-reduced column containing native copper shavings before pressurizing the reactors by adding H_2_ to the specified concentration, replacing the appropriate gas volume, and then pressurizing to 2.5 bar with N_2_. In the case of the 100% H_2_ condition, the headspace was purged with H_2_ for 20 min and was then pressurized to 2.5 bar with H_2_. The reactors were incubated at 38°C statically in the dark.

Reactors were sampled for HS^–^ *via* the methylene blue assay (Fogo and Popowsky, 1949) and Fe(II) *via* the ferrozine assay (Stookey, 1970). Samples for HS^–^ determination were analyzed immediately whereas samples for Fe(II)/(III) determination were first incubated for 16 h in 1N HCl at room temperature (∼20°C) before centrifugation (5,000 × *g*, 5 min, 4°C) to pellet the mineral. The supernatant was split in half, with one half used to measure the concentration of Fe(II). The other half of the sample was used to quantify Fe(III) by first reducing the sample with 0.2 M hydroxylamine hydrochloride for 30 min and then determining total Fe, from which Fe(II) was subtracted to arrive at Fe(III) concentration. Fe(III) was not detected in any incubation experiment.

To further examine the mineral product that forms during reductive dissolution of FeS_2_, 65 μmol of laboratory synthesized nanoparticulate FeS_2_ was incubated in 5 mL (6.5 mM final concentration) in MQ H_2_O undisturbed at 38°C for 24 h under 2.5 bar H_2_ (1.98 × 10^–3^ M aqueous). The pelleted mineral was dried anoxically under a stream of N_2_ gas (∼10 psi delivered through a 22-gauge needle) for ∼2 h while sitting in a warm water bath on low heat to promote drying. The dried mineral was characterized using XRD, as described above, within 2 h of preparation.

### Cultivation Conditions

*Methanosarcina barkeri* strain MS and *M. barkeri* strain Fusaro were obtained from the American Type Culture Collection (ATCC-43569 and ATCC-BAA-2329, respectively). A mutant strain of *M. barkeri* strain Fusaro with deletions in four operons encoding for its five [NiFe]-hydrogenases was constructed as part of a prior study and was used herein (Mand et al., 2018). For all strains, growth medium was prepared without added Fe and S in MQ water and using acid (10% HNO_3_) washed glassware. Specifically, *M. barkeri* was grown in low-salinity medium, as described above for abiotic experiments. The base salts solution was boiled for 10 min then purged with N_2_ gas passed over the heated copper column containing reduced copper shavings for 1 h L^–1^. After sparging, base salts medium was moved to an anaerobic chamber and allowed to cool to room temperature. Once cooled, NaHCO_3_ (2.00 *g* L^–1^) was added, and the pH was adjusted to 7.0 with anoxic 2N HCl. Next, 75 mL of the base salts medium were dispensed into 165 mL serum bottles, sealed with blue butyl stoppers, and capped with aluminum crimp caps. Sealed serum bottles were removed from the anaerobic chamber, the headspace was exchanged for 15 min with N_2_:CO_2_ (80%:20%) gas passed over a heated copper column, and then they were autoclaved. After autoclaving, a sterile and anoxic 100X phosphate solution containing 0.35 *g* L^–1^ K_2_HPO_4_ and 0.23 *g* L^–1^ KH_2_PO_4_ was added to each bottle of base salts medium to a final dilution of 1X. Prior to inoculation, the base salts medium used to cultivate both methanogen strains was amended with 1% (v/v) Wolfe’s vitamins and 1% SL-10 trace metals (no added Fe containing components). All *M. barkeri* cultures were provided with 0.5% (v/v) methanol and 40 mM acetate as a methanogenesis substrates and grown anaerobically with a N_2_:CO_2_ (80%:20%) headspace pressurized to 2.5 bar.

Maintenance cultures were transferred every 4–7 days when they reached late log-phase. Cultures were maintained by providing aqueous sources of Fe and S, which consisted of 20 μM FeCl_2_ and 2 mM HS^–^. The *M. barkeri* Fusaro mutant strain was also provided with 2 mM cysteine to maintain cultures. To inoculate experimental cultures, mid-log phase grown cells were washed by centrifugation (4,696 × *g* for 20 min at 4°C) under anoxic conditions. Spent medium was decanted and washed cells were resuspended in sterile, anoxic base salts medium in a sealed serum bottle. Cultures were handled within an anaerobic chamber throughout the washing procedure. A 10% (v/v) transfer of washed cells was used to inoculate freshly prepared medium. All cultures were grown statically on their sides at 38°C in the dark.

Growth of *M. barkeri* was monitored by quantification of DNA and protein. *M. barkeri* grows in aggregates and strongly associates with FeS_2_ (Supplementary Figure 5), which prohibits accurate cell enumeration. Further, the presence of FeS_2_ minerals in the growth medium interferes with OD measurements, commonly used to quantify growth of *M. barkeri* (Sowers et al., 1993). Therefore, DNA and protein were used as proxies for cell production. In the case of DNA quantification, it is important to note that other *Methanosarcina* species contain fewer genome copies per cell during slow growth compared to fast growth (Hildenbrand et al., 2011) and thus DNA could underestimate biomass production during early and late log phase. It is also not known if cells produce excess EPS or protein during attachment to mineral phases, which could influence protein-based estimates of biomass production. Nonetheless, to quantify DNA, 2 mL of *M. barkeri* MS or Fusaro cultures were centrifuged at 20,000 × *g* for 20 min at 4°C, the supernatant was removed, and the cell pellet was resuspended in a lysis buffer solution containing 489 μL sodium phosphate buffer (MP Biomedicals, Irvine, CA, United States) and 61 μL MT buffer (MP Biomedicals). Once the lysis buffer was added to the cell pellet, the solution was mixed by gentle agitation and subjected to three rounds of freezing at –80°C and heating/thawing at 70°C in a heat block. The mixture was then transferred to a Lysis E tube (MP Biomedicals) and homogenized on a bead beater (Biospec Products, Bartlesville, OK, United States) for 40 s. Finally, the tube and its contents were centrifuged for 15 min at 14,000 × *g* to separate cell debris from DNA. The concentration of DNA in the supernatant was quantified fluorometrically with a Qubit HS Double Stranded DNA kit and Qubit fluorimeter (Invitrogen, Carlsbad, CA, United States). For protein quantification, a 1 mL aliquot of *M. barkeri* cultures was centrifuged at 20,000 × *g* for 20 min at 4°C, the supernatant was removed, and the cell pellet was resuspended in 0.5 M NaOH. The solution was incubated at 99°C in a heat block for 10 min, allowed to cool to room temperature, and then centrifuged for 15 min at 14,000 × *g* to separate debris from protein. The concentration of protein was quantified fluorometrically with a Qubit Protein Assay kit and Qubit fluorometer (Invitrogen, Carlsbad, CA, United States).

Headspace gas from cultures was sampled with a N_2_-flushed syringe to monitor CH_4_ and H_2_ and was diluted with ultra-high purity N_2_ into gas-tight CaliBond bags (Calibrated Instruments Inc., Manhasset, NY, United States). CH_4_ and H_2_ were determined by gas chromatography *via* injection of a 5 mL of sample into an injector valve set at 55°C on an SRI 8610C gas chromatograph (SRI instruments, Torrance, CA, United States). The gas chromatroph was equipped with a 4.5 m × 0.125″ OD Hayesep DB 100/120 packed column with the oven set to 44°C (Valco Instrument Company Inc., Houston, TX, United States). CH_4_ was detected by a flame-ionization detection at 156°C with ultra-high purity He as carrier gas and H_2_ was measured by a pulse-discharge He-ionizer detector at 100°C. Methane and H_2_ peak area values were converted to ppm using pre-mixed gas standards (EGAS Depot, Nampa, ID, United States). Dissolved HS^–^ and headspace CH_4_ measurements were converted to total sulfide and total CH_4_ (dissolved and gas phase) using Henry’s Law.

For dialysis experiments, 100 kDa dialysis tubing was prepared for FeS_2_ and Fe_1–_*_*x*_*S experiments, as previously described (Payne et al., 2021b) using a series of ethanol and MQ H_2_O rinses to wash and sterilize the membranes. Once sterilized, tied dialysis membranes were transferred to an anaerobic chamber where synthetic FeS_2_ (final concentration of 2 mM Fe) or specimen Fe_1–_*_*x*_*S (0.1 *g*) was added to each dialysis bag then the ends were secured using monofilament lines. The same concentrations of FeS_2_ and Fe_1–_*_*x*_*S were added to reactors where minerals were not sequestered in dialysis membranes. The dialysis bags were rinsed with sterile, anoxic MQ H_2_O before transferring to a prepared bottle of medium amended with methanogenesis substrates, trace metals, and vitamins, which was then capped and sealed before removing from the anaerobic chamber. Unamended controls contained dialysis tubing prepared the same as above but without any mineral or Fe added. Upon removing the sealed medium bottles from the anaerobic chamber, the headspace was purged with an 80:20 mixture of 0.2 μm filtered N_2_:CO_2_ for 45 min to remove gas originating from the anaerobic chamber and were then pressurized with N_2_:CO_2_ (final pressure of 1.72 atm) before inoculating with mid-log *M. barkeri* Fusaro wild-type cells grown with sulfide and FeCl_2_ (10% v/v inoculum) that were washed by pelleting cells *via* centrifugation and gently resuspending in anoxic base salts medium within an anaerobic chamber. Anoxic AQDS was added to a final concentration of 2 mM and monitored spectrophotometrically at 325 nm for AQDS and 450 nm for AH_2_QDS.

Cultures of *M. barkeri* MS were grown in quadruplicate on three different sources of Fe and S for RNA-Seq analysis: 2 mM FeS_2_, 20 μM FeCl_2_ and 2 mM L-cysteine, or 20 μM FeCl_2_ and 2 mM Na_2_S. Quadruplicate cultures for each condition were kept separate throughout sample collection, RNA sequencing, and analysis. Cells were harvested at mid-log growth phase, as determined by CH_4_ production, and final biomass was estimated by DNA quantification. To harvest biomass, *M. barkeri* MS cells were subjected to vacuum filtration at low pressure (5 psi) onto 47 mm 0.2-μm Supor 200 PES filters (Pall, Port Washington, NY, United States) within an anaerobic chamber. Using flame-sterilized scissors, each filter was cut in half and each half transferred to a 1.5 mL cryotube that was then sealed before being removed from the anaerobic chamber. The tube and its contents were immediately flash frozen in liquid nitrogen and frozen cells were stored at –80°C until processing.

### RNA Extraction and Transcriptomic Sequencing

Total RNA from *M. barkeri* strain MS was extracted using TRIzol reagent (Invitrogen) following the manufacturer’s protocol with slight modification. Individual frozen half filters containing *M. barkeri* cells were transferred to a Lysis E tube (MP Biomedicals) on ice and 1 mL of TRIzol was immediately added to the frozen filter. TRIzol-treated *M. barkeri* cells were subjected to three cycles of 40 s of bead beating followed by resting for 5 min at room temperature (∼20°C). Two hundred microliter of molecular grade chloroform was added to each tube and each tube was inverted to mix, incubated at room temperature for 3 min, and centrifuged for 15 min at 12,000 × *g* at 4°C. The upper aqueous phase containing RNA was carefully transferred to a clean 2 mL tube. To precipitate RNA, 0.5 mL of pre-chilled (4°C) 100% molecular grade isopropanol was added to each tube and tubes and their contents were incubated on ice for 10 min followed by centrifugation for 10 min at 12,000 × *g* at 4°C. The supernatant was discarded, and 1 mL of 75% molecular grade ethanol was added to wash the RNA. After centrifugation for 5 min at 7,500 × *g* at 4°C, the supernatant was removed, and the RNA pellet was air dried for 5–10 min. Total RNA was dissolved in 50 μL RNA-grade water (Thermo Fisher Scientific, Waltham, MA, United States) by incubating in a 55°C heat block for 10 min. RNA was treated with Turbo DNase (Invitrogen) to remove residual DNA per manufacturer’s instructions. After DNase treatment, the remaining total RNA was subjected to a second round of isopropanol precipitation, ethanol wash, and resuspension as described above. The concentration of total RNA was quantified fluorometrically using the Qubit BR RNA kit and a Qubit fluorometer (Invitrogen) and the quality checked using a NanoDrop ND-1000 spectrophotometer (Thermo Fisher Scientific, Waltham, MA, United States). Absence of DNA was verified by subjecting the RNA extract to 40 cycles of PCR using archaeal-specific 16S rRNA primers (344F/915R) as previously described (Boyd et al., 2013) and checking for amplification products *via* gel electrophoresis. Total RNA was sent to the University of Wisconsin’s Genome Expression Center for quality control, rRNA depletion using custom *M. barkeri* strain MS-specific oligos designed using the sequences for *M. barkeri* MS’s large and small ribosomal subunits, and sequencing on an Illumina NovaSeq 2 × 150 bp.

Paired-end reads were processed using default settings in TrimGalore!,^1^ a wrapper that implements CutAdapt (Martin, 2011) and FastQC^2^ to remove adapter sequences and filter reads, respectively. Reads were aligned to the reference *M. barkeri* MS genome (ASM97002v1) using Bowtie2 (Langmead and Salzberg, 2012). Reads were counted for each locus using HTSeq (Anders et al., 2015) followed by normalization and analysis in DESeq2 (Love et al., 2014) implemented in R v3.6.0.

## Data Availability Statement

The RNA-sequencing data reported in this article have been deposited in the NCBI GEO database (GSE168895).

## Author Contributions

RS and EB conceived and designed the study. RS, DP, ER, and EB collected, interpreted, and analyzed experimental data. GK and WM designed and provided mutant methanogen strains for experiments. RS and EB wrote the manuscript. All authors reviewed the manuscript before submission.

## Conflict of Interest

The authors declare that the research was conducted in the absence of any commercial or financial relationships that could be construed as a potential conflict of interest.

## Publisher’s Note

All claims expressed in this article are solely those of the authors and do not necessarily represent those of their affiliated organizations, or those of the publisher, the editors and the reviewers. Any product that may be evaluated in this article, or claim that may be made by its manufacturer, is not guaranteed or endorsed by the publisher.

## Footnotes

1
https://www.bioinformatics.babraham.ac.uk/projects/trim_galore/

2
https://www.bioinformatics.babraham.ac.uk/projects/fastqc/
