# Supplementary material for: Investigating Abiotic and Biotic Mechanisms of Pyrite Reduction
Source: Front Microbiol. 2022 May 9;13:878387. doi: 10.3389/fmicb.2022.878387 (PMC9124975; doi:10.3389/fmicb.2022.878387)
Supplement: Supplementary file 2 [file Data_Sheet_1.PDF]

Investigating abiotic and biotic mechanisms of pyrite reduction at low temperature

Rachel L. Spietz[1], Devon Payne[1], Gargi Kulkarni[2], William W. Metcalf[2], Eric E. Roden[3], and Eric S. Boyd[1]

Supplementary Online Information

Supplemental Materials and Methods

***Electron Microscopy*.** Mid-log phase *M. barkeri* strain Fusaro wild-type cells were fixed in 2.5% electron microscopy grade glutaraldehyde (Electron Microscopy Sciences, Hatfield, PA) for four hours at room temperature. Fixed cells were applied to a Au-sputtered 0.2 µm polycarbonate filter (MilliporeSigma, Burlington, MA) by low-pressure vacuum filtration. Cells were dehydrated by an ethanol series (50%, 70%, 80%, and 95%) passed through the filter at low pressure. Images were collected in the Imaging and Chemical Analysis Laboratory at Montana State University using a high-resolution FE-SEM (Supra 55VP, Zeiss, Thornwood, NY) with a primary electron beam energy of 1 keV at different magnifications as previously described (1).

Supplemental Figures

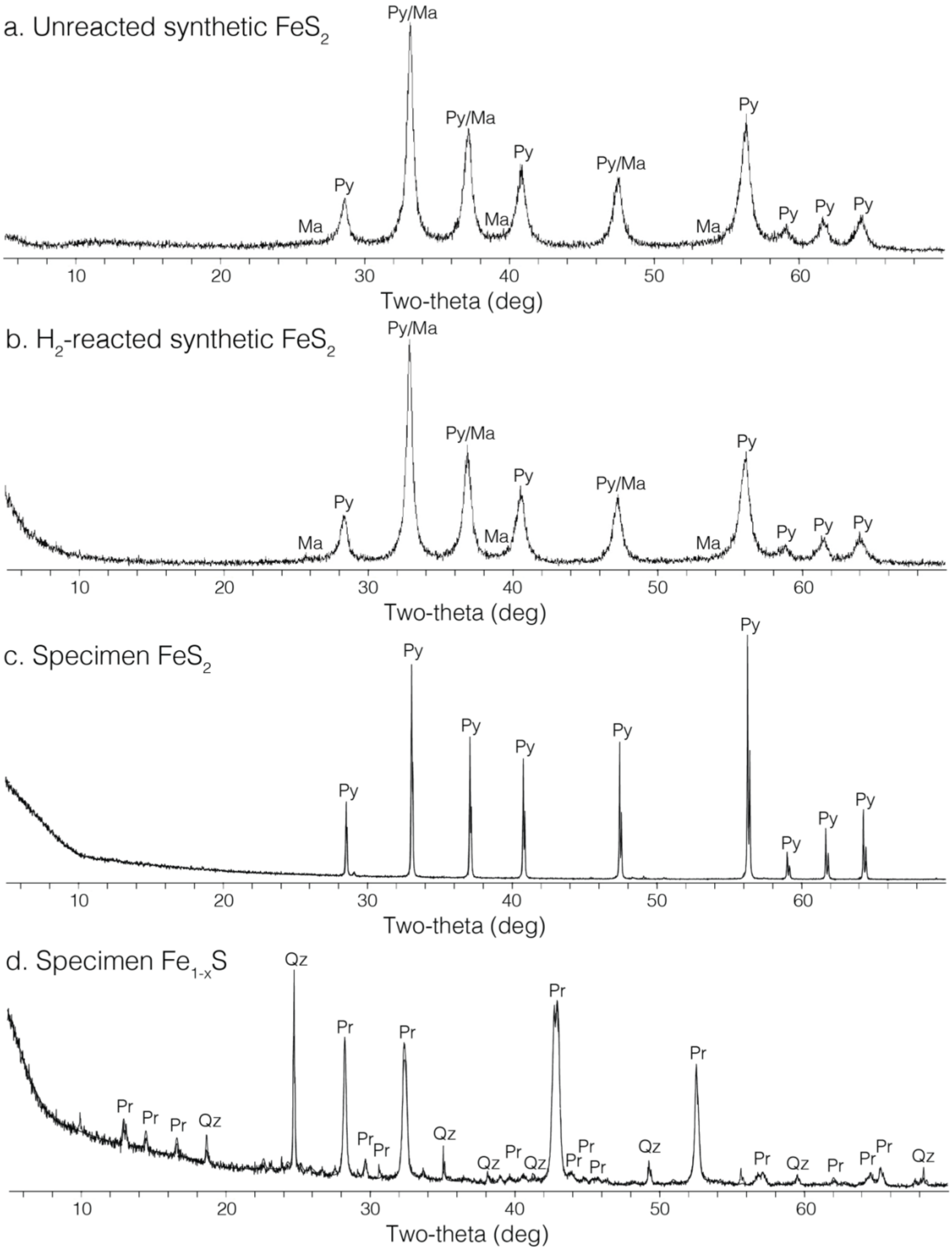

Figure S1. X-ray diffraction (XRD) analysis of minerals used in this study. Minerals were dried under a stream of nitrogen (N_2) gas, powdered, and subjected to XRD analysis. In panel (**a**)

unreacted synthetic nanoparticulate pyrite (FeS_2) slurry used in growth and abiotic experiments contained 97.4 % pyrite (Py) and 2.6 % marcasite (Ma) peaks by weight. In (**b**) the synthetic FeS_2 was reacted in a sealed serum bottle with 3.4 bar H_2 and showed a nearly identical spectra as (**a**) with 96.5% pyrite (Py) and 3.5 % marcasite (Ma) by weight. The specimen FeS_2 (**c**) and specimen pyrrhotite ($Fe_{1-x}S$) (**d**), used in growth and abiotic experiments, were powdered and washed prior to drying under N_2 then subjected to XRD analyses. Specimen FeS_2 contained 100% pyrite (Py) and specimen $Fe_{1-x}S$ contained 78.9% pyrrhotite (Pr) and 21.1% quartz (Qz).

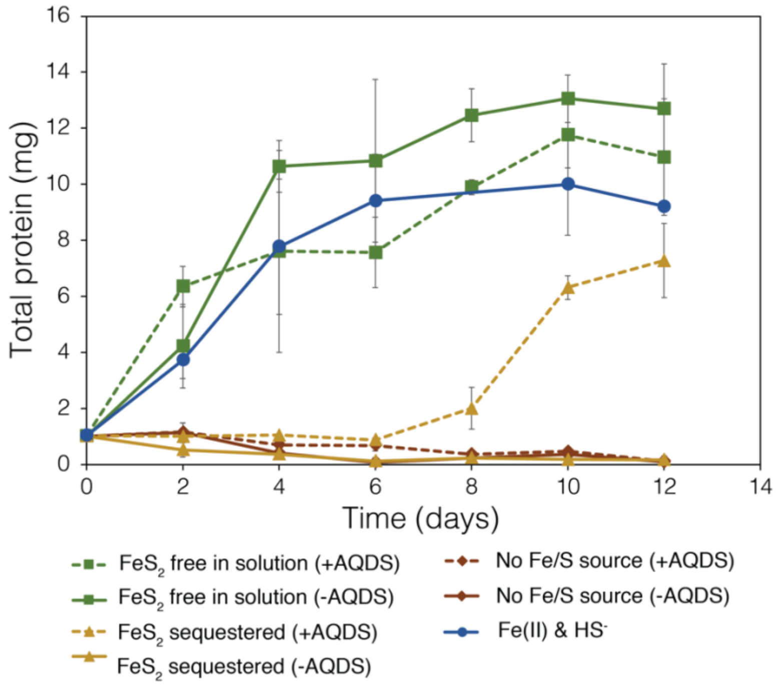

Figure S2. Production of protein by *Methanosarcina barkeri* strain Fusaro when grown with pyrite (FeS_2) free in solution or sequestered in 100 kDa dialysis tubing to prevent physical association. Negative control cultures contained no added Fe or S source, while positive control cultures were provided with 20 µM Fe(II) and 2 mM HS^-. AQDS was provided at a final concentration of 20 mM. Methanol and acetate were provided as the methanogenesis substrates and carbon sources for all conditions tested. Averages were calculated for triplicate cultures and standard deviations for the triplicates are shown. Experiments were conducted in 165 mL serum bottles containing 75 mL medium. These data accompany growth kinetics and activities presented in **Figure 6**.

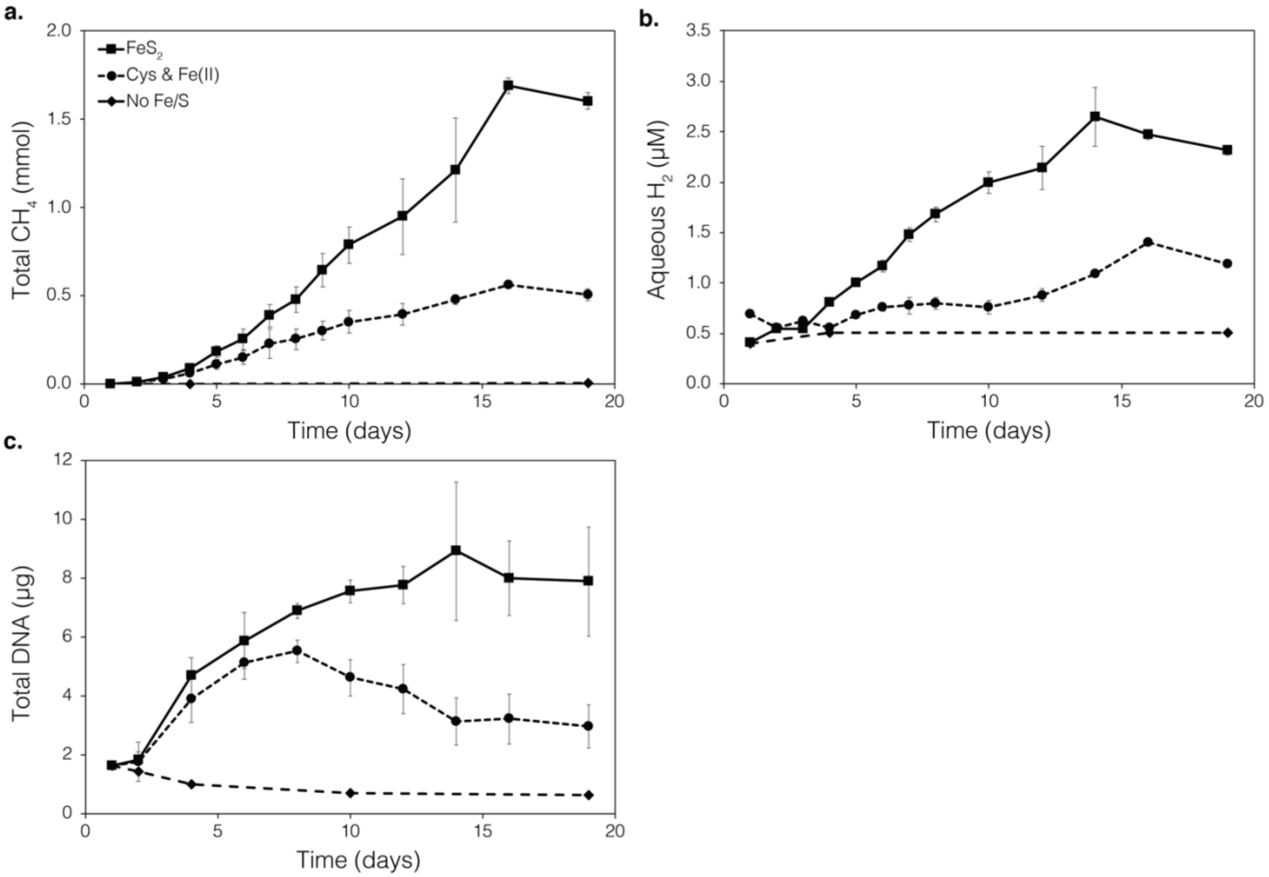

Figure S3. Production of (**a**) methane (CH_4), (**b**) hydrogen (H_2), and (**c**) biomass (DNA) during growth of *Methanosarcina barkeri* strain MS when provided with 2 mM synthetic pyrite (FeS_2) nanoparticles or 20 µM ferrous iron (Fe(II)) and 2 mM cysteine (Cys) as sole iron and sulfur source. Methanol and acetate were provided as methanogenesis substrates and carbon sources for all conditions tested.

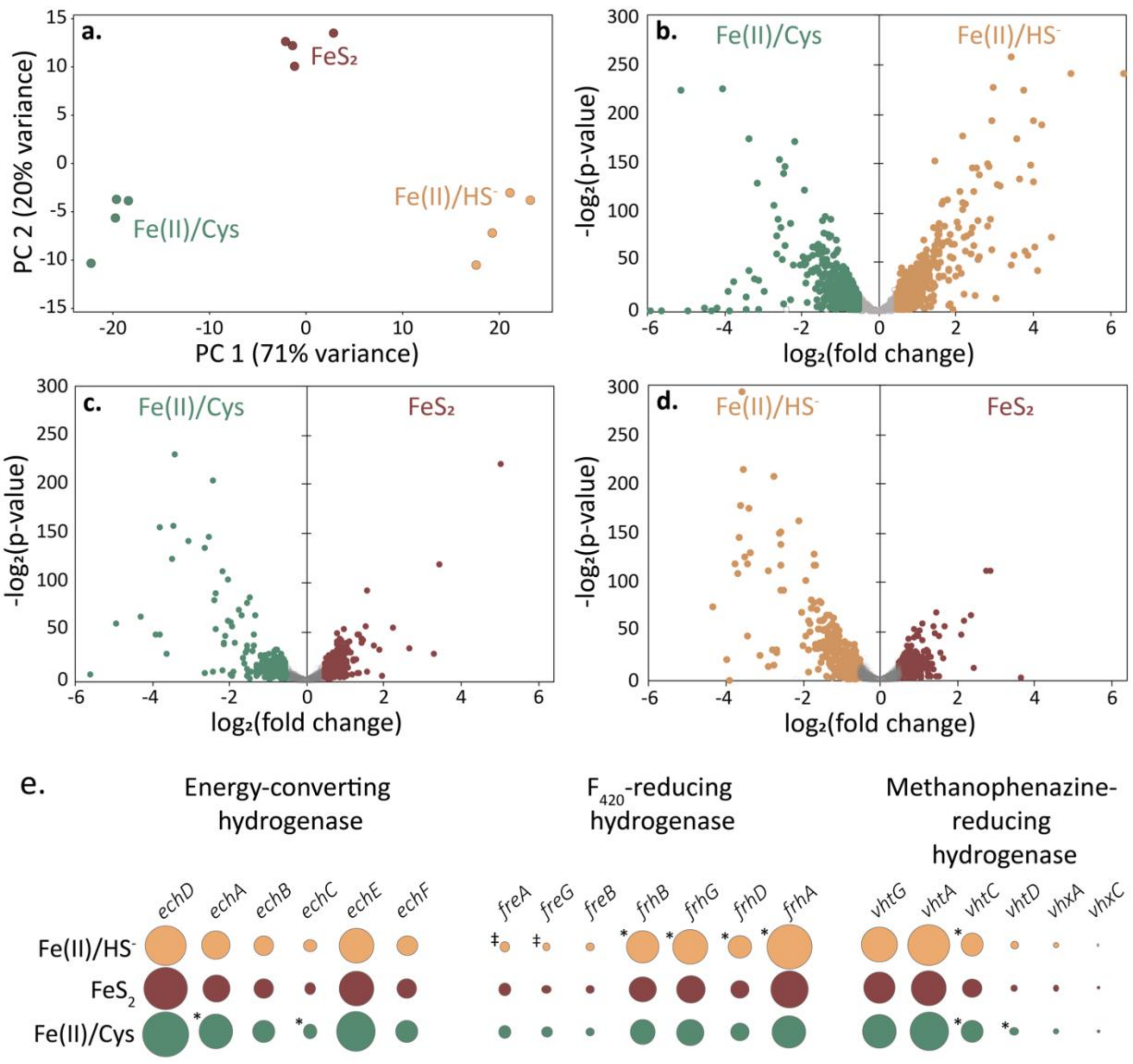

Figure S4. Global view of *Methanosarcina barkeri* MS genes that were differentially expressed when provided with different sources of iron (Fe) and sulfur (S), as specified in each figure panel. (**a**) Principal component analysis (PCA) was used to determine the level of variation among replicates within treatment groups and across treatment groups. (**b-d**) Plot of the pairwise log_2 fold change (LFC) in the expression of each gene (depicted by a single dot) as a function of the $-log_{10}$ of the adjusted p-value ($-log_{10}$(p-value)). Points that are in color rather than grey specifies protein transcripts that are significantly different above an adjusted p-value of 0.05 and that have a LFC that is either greater or less than 0.5. Points that are significantly up-regulated in the pyrite (FeS_2) growth condition relative to both ferrous iron (Fe(II))/cysteine (Cys) (**c**) and Fe(II)/sulfide (HS^-) (**d**) growth conditions are plotted in **Figure S2**. (**e**) Expression of [NiFe]-hydrogenases from *M. barkeri* MS cells grown with either FeS_2 or Fe(II)/Cys as the sole Fe and S source. Methanol and acetate were provided as methanogenesis substrates and carbon sources for all conditions tested. Cultures grown with FeS_2 contained 83 mL of base salts medium and 87 mL headspace; cultures grown with Fe(II) and Cys contained 83.75 mL of base salts medium and

86.25 mL headspace. In (**c**) expression of five [NiFe]-hydrogenase gene operons (1 energy converting (Ech), 2 F_{420}-reducing (Frh, second copy labeled as Fre), 2 methanophenazine-reducing (Vht, second copy labeled as Vxt)) involved in intracellular hydrogen production in *Methanosarcina barkeri* MS during growth on either FeS_2 or Fe(II) and cysteine. The sizes of the bubbles are proportional to the normalized expression of transcripts detected for each specified gene and have been standardized within each operon. These hydrogenases are homologous to the same five hydrogenases that were knocked out in the [NiFe]-hydrogenase mutant strain of *M. barkeri* Fusaro (*see* **Figure 4**). Genes that were significantly ($p < 0.05$) upregulated ($\log_2$ fold change (LFC) > 0.5) are marked with a *. Gene loci, normalized transcript expression values, and LFC with associated adjusted *p*-values are provided in **Table S1**. Abbreviations: Fe(II), ferrous iron; Cys, cysteine.

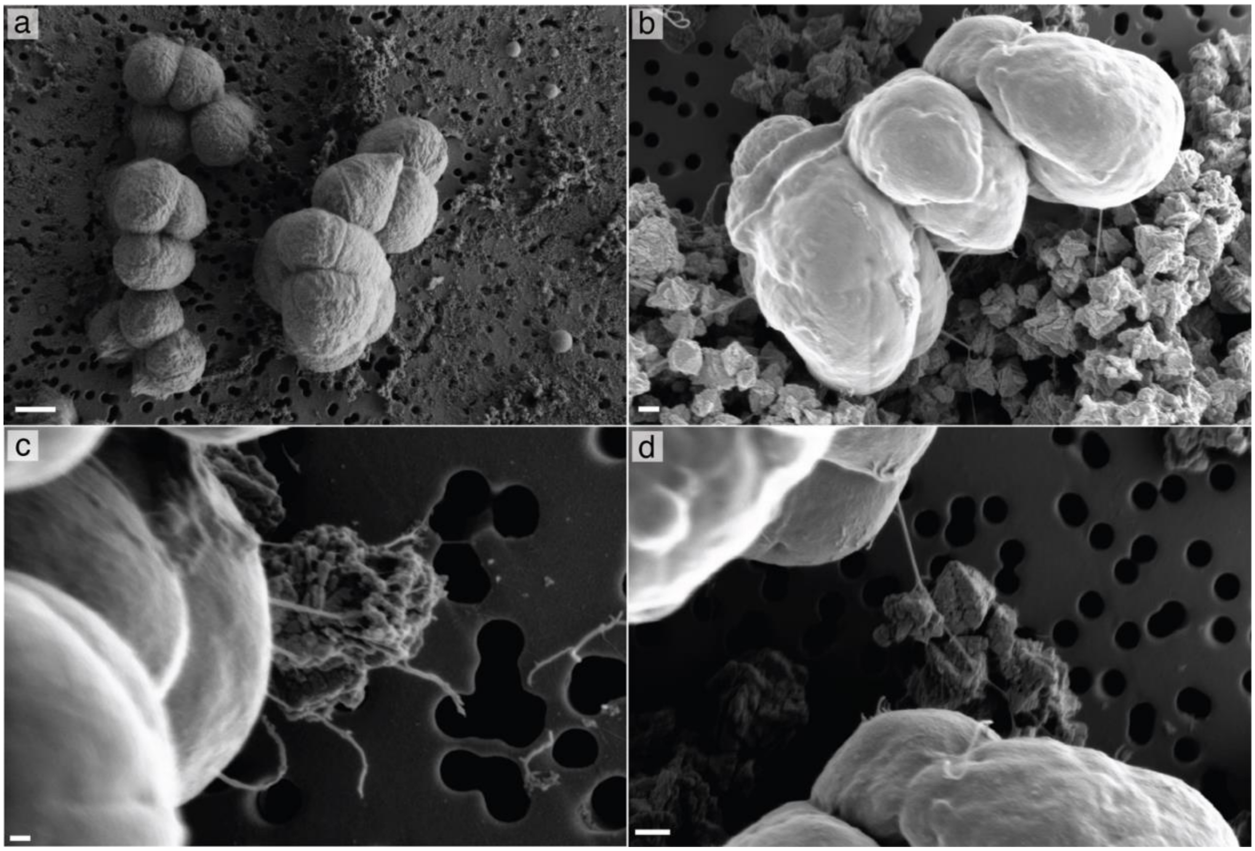

Figure S5. Field emission scanning electron micrographs of *Methanosarcina barkeri* Fusaro wild-type grown with different sources of iron and sulfur. (**a**) Cells grown with cysteine and ferrous iron did not associate with solid-phase precipitates. When grown with synthetic pyrite (FeS_2) nanoparticles as the sole source of iron and sulfur (**b**-**d**), *M. barkeri* Fusaro cells associated with FeS_2 nanoparticles via what appear to be dehydrated strands of extracellular polymeric substances (EPS) (indicated by white arrows in **c** and **d**). Cells in all conditions were grown with methanol and acetate as methanogenesis substrates and carbon sources. Scale bars: (**a**) 1 µm, (**b**, **d**) 300 nm, and (**c**) 100 nm.
